# Women Experts and Gender Bias in Political Media

**DOI:** 10.1093/poq/nfad011

**Published:** 2023-05-11

**Authors:** Adam L Ozer

**Affiliations:** Research Associate, Department of Political Science, Machine-Assisted Human Decision-Making (MAHD) Lab, University of Houston, Houston, TX, US; and Professional Affiliate, Department of Government, Electoral Psychology Observatory, London School of Economics, London, UK

## Abstract

Widely held gender stereotypes present obstacles for women experts, who are generally evaluated less positively than equally qualified men across a range of fields. While audiences may view women as better equipped to handle certain feminine-stereotyped issues, Role Congruency Theory suggests that expert authority in politics may be incongruent with traditional feminine gender roles, leading to a subsequent backlash. Building upon the latter theory, I hypothesize that when cued to consider the expertise of a news source, the (in)congruence of gender-stereotyped roles will activate gender biases which increase the gap in evaluations and trust of women and men. Using selection experiments, I assess the relationship between domain-relevant expertise and gender biases across a range of gender-stereotyped issues. I find that women experts are rewarded less for additional expertise and punished more severely for a lack of expertise, exacerbating gender-based biases relative to the control. I find that this pattern is consistent across both masculine- and feminine-stereotyped issues, including issues that disproportionately impact women, such as women’s health care and the gender wage gap. The addition of competing partisan cues, however, overwhelms the influence of gender. The normative implications suggest women in the media often face an uphill battle to advocate for their interests on key issues that affect them even when they may have more direct relevant experience in addition to their qualifications.

*“Friends and colleagues—both male and female—warned me that making this speech would harm my career by instantly typecasting me as a female COO and not a real business executive.” – Sheryl Sandberg, COO of Facebook, at TEDWomen 2010* ^1^

Women experts are substantially underrepresented in United States political news media. According to the Global Media Monitoring Project in 2015, only 36 percent of news stories featured women experts. This disparity was even larger for political news, which cited women experts 21 percent of the time (World Association for Christian Communication 2015). While news agencies have argued that it is difficult to find a reliable supply of women experts, research shows that this is heavily influenced by a general skepticism toward the qualifications of expert women. Individuals have been shown to provide more scrutiny toward women’s qualifications (Ditonto, Hamilton, and Redlawsk 2014). In addition, women experts in the sciences and academy are viewed as less qualified and are subsequently hired less often relative to identical men (Reuben, Sapienza, and Zingales 2014; Quadlin 2018). This research shows a concerning pattern with regard to the intersection between expertise and gender cues.

This societal perception of women as holding less expertise relative to equally qualified men holds important and concerning implications for the ways in which Americans consume political news. While Americans tend not to have a highly detailed knowledge of politics and current events, they can rely on experts to help synthesize information to help them make semi-informed decisions (Downs 1957; Popkin 1994; Lupia and McCubbins 1998; Lupia 2013). Individuals find expert news sources more persuasive and tend to select expert sources at a higher rate, allowing experts to help guide the audience to better-informed decisions, even in highly polarized political environments (Druckman 2001; Boudreau and McCubbins 2010; Ozer 2020). Yet, if women are perceived to lack expertise relative to men, to what degree do gender cues and roles present an obstacle for women experts in political media when attempting to disseminate information to their audience? What are the implications if women experts face additional hurdles when trying to effectively communicate with the audience when discussing important political issues?

In this research, I seek to directly test the effect of simultaneous expertise and gender cues in political media. Citing Role Congruency Theory, I argue that cues highlighting expertise exacerbate implicit gender biases, increasing the gap between men and women in perceived credibility and news consumption. Leveraging two selection experiment designs, I show that women are rewarded less for high levels of expertise and punished more severely for a lack of expertise relative to identical men. This increases the gender gap in perceived credibility and news consumption. This effect is consistent for both men and women respondents and across a variety of masculine- and feminine-stereotyped political issues, including policies which disproportionately impact women. Yet, while this gap between men and women experts is substantial, its impact is nearly completely overwhelmed by competing partisan cues which trigger polarization. This suggests that while the individuals may be willing to overlook their initial skepticism toward women in political media, they tend to do so only for partisan ends. These results hold important implications for women experts in politics, as they face an uphill battle to share their perspective, particularly on issues that impact women the most.

## The Role of Experts in Political Media

Individuals can turn to credible experts, often through the media, to leverage their more knowledgeable perspective to economize and ease the cognitive burden of seeking accurate political information (Downs 1957; Popkin 1994; Lupia and McCubbins 1998; Lupia 2013). Expertise itself can be loosely defined as the assessment of the speaker’s qualifications, intelligence, and competence (Boudreau and McCubbins 2010; Lupia 2016). In this sense, expertise is relative and contextual, meaning that individuals ought to value information from sources that can guide the listener toward the most sensible option, and the degree to which a specific cue informs this perception may differ among individuals (Lupia and McCubbins 1998; Boudreau and McCubbins 2010). For example, while one individual may view “political scientist” to be a cue indicating expertise, another may view that individual to be lacking real-world experience due to perceptions of the proverbial ivory tower. It is worth noting that the assumption that expert advice leads to better decision-making is lacking in nuance and generally overlooks vital questions regarding political expertise and citizen competence (Kuklinski et al. 2001). Following the advice of experts does not guarantee better outcomes. In certain contexts, such as forecasting important future events, evidence suggests that expert pundits perform poorly, often performing about as well as a coin flip (Tetlock 2017). Moreover, expertise does not guarantee that the expert would not deliberately mislead the audience due to dubious character or conflict of interests. Nonetheless, in the broadest sense, experts are assumed to be more capable of producing well-researched perspectives that help individuals synthesize and interpret complex political information, making their input an important part of healthy democratic discourse.

However, the political media landscape is often oversaturated with voices with varying levels of expertise. A 2016 *Washington Post* analysis found that in just an eight-day period, 601 pundits made an appearance on the three major cable news networks, with up to 11 on screen at once (Farhi 2016). Perhaps more normatively distressing, 42 percent of news reports that feature an expert perspective juxtapose that expert with a nonexpert political source (Merkley 2020). The presence of multiple sources, qualified and unqualified, can lead individuals to misplace trust in less reliable sources and lead to worse overall decision-making (Boudreau 2013).

With so many competing voices, how do individuals distinguish between expert and nonexpert sources? Media outlets are able to prevent some audience confusion through the use of source expertise cues—subtle but direct signals that individuals can interpret to assess the qualifications and expertise of an information source (Hovland and Weiss 1951; Belknap 1954; Giffin 1967; Lupia 2013). By establishing expert authority through leveraging minor source cues, expert sources can increase their persuasiveness and lead individuals to more informed decisions (Druckman 2001; Boudreau and McCubbins 2010). Prior literature suggests that expertise cues can be quite useful in facilitating effective communication between experts and individuals, often yielding the desired increase in information-seeking, evaluation, and persuasion on polarizing political topics despite partisan biases (Bullock 2011; Boudreau and MacKenzie 2014, 2018; Schuldt and Roh 2014; Bolsen and Druckman 2015; Jang and Hart 2015; Ozer 2020). Yet, individual assessments of expertise are not driven by a single cue. Instead, individuals often assess the expert credibility of a source based on a number of competing cues, including the source’s occupation, partisanship, and gender (Lupia and McCubbins 1998). Thus, when studying the effect of expertise in the political media sphere, it is vital to consider the interplay of various cues and issue contexts.

## Women, Politics, and Perceived Expertise

Much of the difficulty that women face in terms of claiming their status as authoritative experts lies within the stereotyped traits and societal roles for both men and women. Role Congruency Theory dictates that stereotypical traits assigned to women and men are born out of their separate historical societal roles (Eagly and Karau 2002). Women and femininity are stereotyped as nurturing and compassionate, with women placed into more supportive societal roles. Comparatively, men and masculinity are stereotyped as tough and assertive, traits more conducive with leadership roles. The impact that these roles have in the political sphere is context dependent, with women often viewed as better equipped for care-based female stereotyped issues, like health care and equality, and less well equipped to tackle conflict-oriented male stereotyped issues, like foreign policy and national security (Huddy and Terkildsen 1993).

Yet, across a range of masculine- or feminine-dominated issues, the role of an expert requires that the expert in question speak from a position of qualified authority, which individuals may associate with masculine traits. As a result, past evidence alludes to women leveraging expert authority being viewed as violating the traditional gender roles outlined in Role Congruency Theory, potentially undermining their credibility and persuasiveness. Individuals are often more skeptical of women’s expertise, more actively searching for and carefully vetting the qualifications of women candidates relative to identical men (Ditonto, Hamilton, and Redlawsk 2014). This contributes to an overall perception that women are “warm, but dumb,” viewed as more sympathetic than men, but less competent and lacking domain-relevant expertise (Fiske 2012). Individuals show a strong tendency to fall back upon these prevailing gender stereotypes in the political sphere, resulting in lower evaluations for female politicians in high-threat contexts (Simas 2020). This suggests that women experts may face an uphill battle, with their own expertise reinforcing stereotyped gender roles that put them at a disadvantage relative to equally qualified expert men.

Issue-based stereotypes may hold influence over gender biases in evaluation and behavior as well. Yet, the presence of direct gender cues (e.g., a media expert that clearly identifies as a woman) tends to activate gender-based stereotypes to a much greater degree than the issues themselves, resulting in stronger negative evaluations toward women that violate these stereotyped roles (Bauer 2020). This contributes to a general pattern in which women experts are evaluated less positively than identical men across a range of masculine- and feminine-stereotyped issues. For example, women scientists and academics are often viewed as less qualified and hired less often than identical men (Reuben, Sapienza, and Zingales 2014; Quadlin 2018). Similarly, individuals are more supportive of identical diplomatic proposals when put forward by expert men relative to equally qualified women (Anisman-Razin, Kark, and Saguy 2018). As a result, women are often punished more severely for mistakes and misdeeds. For example, individuals punish women politicians more severely than identical men when they catch that politician in a lie (Pereira 2020; Simas and Murdoch 2020).

Further, the relationship between gender roles and political expertise is impacted by partisan polarization in the political environment in complex and often contrasting ways. On one hand, partisan polarization has been shown to exacerbate gender-based stereotypes and biases. Klar (2018) finds that polarization has not only increased hostility toward the out-party, but has overall increased distrust toward women in politics, even among women respondents. Klar finds that this phenomenon is driven primarily by increasing polarization regarding the concept of feminism, as well as partisan-influenced anti-feminist attitudes. This suggests that polarization negatively impacts perceptions of women in politics in an asymmetric fashion. This is underscored in trends regarding recent high-profile women in politics. For example, while belief that women face discrimination in the workplace increased support for Hillary Clinton in 2008 among Democratic Party activists, this belief was negatively correlated with support for Sarah Palin, implying that support for female candidates mirrors the polarized ideological beliefs of the candidates themselves (Sharrow et al. 2016).

Relatedly, evidence shows that Democratic political figures tend to leverage feminine rhetorical style and mention feminine-stereotyped issues, while Republicans leverage a masculine rhetorical style more often (Bystrom and Hennings 2013). These more feminine political rhetorical styles are perceived as more liberal irrespective of the speaker’s gender, with Democratic (Republican) respondents showing a preference (distaste) for feminine speech (Roberts and Utych 2022). This phenomenon is even reflected in perceptions of the Democratic candidates in the 2016 election. Respondents assigned more masculine traits to Hillary Clinton and more feminine traits to her more left-wing progressive opponent Bernie Sanders, with greater perceptions of masculinity for Clinton correlating with less support as predicted by Role Congruence Theory (Conroy, Martin, and Nalder 2020). The impact of these trends is also felt asymmetrically based on partisan preferences. Simas and Murdoch (2020) find that women in politics are punished more severely than men for equivalent scandals, but this effect is exclusive to the out-party. For example, the authors find that individuals punish women in politics more harshly than men when caught in a lie, but only if the politician in question is a member of the out-party, while displaying more leniency toward women in the in-party. Thus, it is possible that the presence of women in political debates or the mention of key women’s issues may serve to trigger both gender-based and partisan-based reactions that may serve to increase both polarization and gender biases.

On the other hand, contrasting evidence suggests that partisan polarization is so strong that it may overwhelm gender cues and stereotypes, leading to more egalitarian (albeit hyper-partisan) opinions, perceptions, and behavior. Recent evidence from United States congressional elections shows that while gender stereotypes and cues impact voter perceptions, they have little substantive impact on vote choice, with decisions heavily dominated by partisanship (Dolan 2014). Candidate partisanship and subsequent trends in polarization also appear to dominate voters’ considerations of gendered policy-based stereotypes (Dolan and Lynch 2016). This is broadly consistent with similar works showing that partisan preferences and polarization dominate political opinion and behavior, overwhelming the influence of competing considerations such as policy position (Cohen 2003; Achen and Bartels 2017) and candidate characteristics (Goren 2002; Simas and Ozer 2017). This suggests that while individuals may prefer a man to a woman as a source of political information, they may be willing to overlook this preference should that woman (man) be a member of the out- (in-)party; technically more egalitarian, but hardly normatively encouraging.

Finally, while the aforementioned literature establishes trait-based and issue-based gender biases in polarized and non-polarized contexts, many of these works do not seek to disentangle the trait and issue-based factors that may be driving these individual gender biases. In perhaps the best approach to these questions, Anisman-Razin, Kark, and Saguy (2018) manipulate both gender and expertise-based cues, finding that only men gain in persuasiveness based on expertise in diplomatic and strategic negotiations. However, this study is intentionally limited in scope, focusing narrowly on Israeli-Palestinian foreign policy with a sample composed exclusively of Israeli undergraduates. While this serves the authors’ purposes well, the masculine-stereotyped issue context of foreign policy conflict does not address whether the gap in evaluations between men and women is driven by trait-driven gender biases regarding expertise or issue-based gender stereotypes. Moreover, it is unclear how well these results apply to a media context. Through the use of selection experiment designs, I seek to assess the relationship between domain-relevant expertise and gender biases across a range of gender-stereotyped issues. I forward three hypotheses. First, in line with past literature, I expect that individuals find news sources with higher levels of expertise to be more persuasive (Druckman 2001; Ozer 2020). While individuals may hold unequal perceptions of identical woman and man experts, I expect that individuals will trust a high (low) expertise source more (less) irrespective of the source’s gender. More formally:

**Expertise Hypothesis (H1):** Individuals will select a high-expertise source more and low-expertise sources less irrespective of gender.

Second, I anticipate that individuals will trust women in the news less than men. Similar to the prior literature, which shows that women are often viewed as less knowledgeable than equally qualified men in their field (Reuben, Sapienza, and Zingales 2014; Quadlin 2018), I expect respondents will view women sources as less credible than men and trust women less overall. More formally:

**Gender Gap Hypothesis (H2):** Individuals will find women less credible than men and will select women in the news less than men.

Third, and most pertinent to this theory, I anticipate that the role of an authoritative expert is one that is masculine stereotyped irrespective of the gendered nature of the issue context. As such, source cues that signal levels of expertise to the audience may activate feminine stereotypes that increase gender biases. Women that assume the role of an expert violate these gender congruency norms, causing a backlash that undermines the additional persuasiveness exhibited by expert sources. Similarly, cues that indicate a lack of expertise should lead individuals to discount the perspective of a nonexpert woman to a larger degree than a nonexpert man. In both cases, as individuals are cued to consider the expertise of the news source, the (in)congruency of gender-stereotyped roles will activate gender biases which increase the gap in evaluations and trust of men and women. More formally:

**Women Experts Hypothesis (H3):** Cues which signal high or low source expertise activate gender stereotypes and bias, increasing the gap in perceived credibility and selection between identical men and women.

## Study 1

### Study Design

I leveraged a census-matched sample of 525 respondents from Lucid, an online survey service. The fieldwork was completed October 19–21, 2019 (the sample was 53 percent female, 74 percent white, median age 45, 55 percent Democrat/lean Democrat, 34 percent Republican/lean Republican). Lucid is a low-cost service that utilizes census-matching to ensure that the samples gathered are representative of the United States population. Experimental results from Lucid samples replicate in the vast majority of instances on other, more traditional representative samples (Coppock and McClellan 2019).^2^

Respondents participated in a selection experiment, viewing several sets of (fabricated) headlines on salient political topics in a within-subjects design. Selection experiments, in which respondents are presented with two headlines or articles and asked to select which they prefer, have been an invaluable tool in extensive works on partisan selective exposure (e.g., Stroud 2011; Feldman et al. 2013, 2018). Such repeated measure designs have been shown to increase precision without altering substantive findings (Clifford, Sheagley, and Piston 2021). In each set of headlines, one author argued in favor of a certain policy (pro-author) while the other argued against that policy (con-author). This specific design is meant to closely mimic how many Americans would receive news on online social media platforms like Facebook or Twitter, with the competing article headlines being akin to different news stories available on one’s typical social media newsfeed or on a social media trending/homepage. While this design mirrors online news media, it is also similar to television news formats, with two pundits arguing for different perspectives on a political issue. Each headline featured a short byline introducing the author, similar to a news article byline or television chyron. After viewing the headlines, respondents selected which of the two headlines they preferred and answered a brief battery of questions before moving on to the next set of headlines.^3^

The headlines discussed six political issues: 1**)** the health care birth control mandate (birth control), 2**)** paid parental leave (parental leave), 3**)** gender-based wage discrimination (wage), 4**)** automatic voter registration, 5**)** military drone strikes on foreign combatants (drone strikes), and 6**)** international trade tariffs (tariffs). These issues provide a contrast in gender stereotypes, with the former three issue frames not only representing traditional feminine-stereotyped topics (e.g., health care), but also representing issues with policy repercussions that disproportionately impact women.

For each set of headlines, I manipulated the author bylines to provide cues indicating the authors’ levels of expertise. I excluded photos to prevent confounds from race or attractiveness. For each set of headlines, one author was randomly assigned a high-expertise cue while the other received a low-expertise cue.^4^ The expertise manipulations are unique to each issue frame (table 1). I ran a series of manipulation checks to ensure that each expertise cue was not confounded by perceptions of political ideology (see Supplementary Material D). Manipulation tests reveal that the high and low-expertise manipulations functioned as intended, with high-expertise authors perceived to have greater expertise. This does not indicate that low-expertise authors have no expertise whatsoever. Rather, the manipulation tests indicate that high-expertise authors have a greater degree of expertise *relative* to low-expertise authors. In addition, I added a control condition in which the expertise of both authors was held constant with no mention of expertise (authors were referred to as “contributors” with no mention of occupation). This serves as a general baseline to compare the effects of changes in expertise. I manipulated the gender of the pro-author via a subtle change in the authors’ names (e.g., Ryan Frank became Rebecca Frank). These names were selected using a random name generator for generic Anglo-sounding names. I created three experimental conditions: 1**)** a woman pro-author debating a man con-author (woman pro-author), 2**)** a man pro-author debating a woman con-author (man pro-author), 3**)** a control condition in which both authors are men to be utilized as a comparison (control). The end result is a 3 x 3 experiment **(**high/low/control expertise x control/man/woman pro-author). Each of the 525 respondents viewed and assessed 6 sets of headlines, bringing the total number of observations to 3,150 (see table 2 for potential treatment combinations).

**Table 1.** Manipulations by issue.

Issue frame	High-expertise cues	Low-expertise cues
Birth control	Medical doctor	Journalist
Parental leave	Economist at the Bureau of Labor Statistics	Journalist
Wage	Economist and business consultant	Journalist
Automatic voter registration	F.E.C. senior elections lawyer	F.E.C. legal clerk
Drone strikes	C.I.A. counterintelligence strategist	United States Embassy language translator
Trade tariffs	Assistant to the United States Trade Representative	Department of Transportation clerk

**Table 2.** Manipulation combinations (Study 1).

	Pro-author	Con-author
1	High-expertise woman	Low-expertise man
2	High-expertise man	Low-expertise woman
3	High-expertise man	Low-expertise man
4	Low-expertise woman	High-expertise man
5	Low-expertise man	High-expertise woman
6	Low-expertise man	High-expertise man
7	Woman (no expertise cue)	Man (no expertise cue)
8	Man (no expertise cue)	Woman (no expertise cue)
9	Man (no expertise cue)	Man (no expertise cue)

### Measures

The main independent variables are the experimental treatments assigned to the pro-author.

For the expertise manipulation, I leverage binary measures which indicate which treatment the respondent received: high-expertise pro-author, low-expertise pro-author, or control. Similarly, I use binary measures to indicate the assignment of the gender treatment: woman pro-author, man pro-author, or control.

The main dependent variable for this analysis is article selection, which represents individual choice in news consumption. Respondents selected which of the two articles they would prefer to read if given the choice (1 = pro-author, 0 = con-author).^5^ As a preliminary measure, respondents also indicated which of the two authors they believe has more expert credibility (1 = pro-author, 0 = con-author). I included this measure as a means to ensure that potential changes in selection correlate with changes in perceived credibility. The credibility measure is highly correlated with the choice measure (r = *.*61).

## Preliminary Analyses

Table 3 displays the mean perceived credibility and selection of pro-authors based on expertise cues. I collapsed across the gender conditions and leveraged simple difference-of-means tests to gauge the statistical difference between authors based on their expertise.

**Table 3.** Credibility and selection based on author expertise.

	Percentage saying that the pro-author is credible %	Percentage that selected the pro-author argument %
High-expertise pro-author (*n* = 1,018)	61.5 (*p* = .001)	62.2 (*p* = .001)
Control pro-author (*n* = 1,024)	51.1	53.5
Low-expertise pro-author (*n* = 1,085)	36.3 (*p* = .001)	44.1 (*p* = .001)

*Note:* *P*-values represent statistical difference from control pro-author with *p*-values reflecting two-tailed tests. Full regression table for left column from which predicted probabilities were derived can be found in Supplementary Material table B1. Full regression table for right column can be found in Supplementary Material table B2.

Preliminary results support the Expertise Hypothesis. Individuals find high-expertise sources to be more credible relative to the control. Similarly, respondents perceive low-expertise sources to be less credible relative to the control with no expertise cues. This indicates that the manipulations function as intended. Similarly, respondents select high-expertise sources 8.7 percent more often and select low-expertise sources 9.4 percent less often relative to the control. While intuitive, these results demonstrate the influence of relatively subtle expertise cues and support H1.

Table 4 presents similar difference-of-means testing based on the gender of the pro-author, collapsing across the expertise dimension. These preliminary results support the Gender Gap Hypothesis (H2). All else held equal, woman news sources are viewed to be 6.2 percent less credible than equivalent men. This finding matches previous works showing that women in intellectual fields are generally perceived to have less expertise relative to equally qualified men. Similarly, respondents select women 3.3 percent less often than men. While this latter effect is small, the cue itself—a simple change in the author’s name with no visual indicator of author gender presentation—is enough to substantially and negatively impact respondent perception and behavior.

**Table 4.** Credibility and selection based on author gender.

	Percentage saying that the pro-author is credible %	Percentage that selected the pro-author argument %
Man pro-author (*n* = 1,073)	51.8	55.1
Woman pro-author (*n* = 1,055)	45.6	51.8
Gender gap (difference)	6.2 (*p* = .001)	3.3 (*p* = .001)

*Note:* *P*-values represent statistical difference in the gender gap. Full regression table for left column from which predicted probabilities were derived can be found in Supplementary Material table B1. Full regression table for right column can be found in Supplementary Material table B2.

### Analysis

While preliminary analysis offers a good starting point to test the Women Experts Hypothesis (H3), I generated predicted probabilities (table 5) for the likelihood that the respondent selects the pro-author. I generated these probabilities via within-subjects logistic regression analyses, including dichotomous indicators of the treatment received, a fixed effect for the issue frame, and a random effect for the respondent. A full regression table can be found in Supplementary Material B (table B1).^6^ A similar analysis featuring the perceived credibility dependent variable yields highly similar results and has been placed in Supplementary Material B (table B2) for parsimony.

**Table 5.** News article selection based on author expertise and gender.

	Percentage that selected woman pro-author		Percentage that selected man pro-author		Gender gap (difference)	
	%	*n*	%	*n*	Gap, %	(*p*-value)
Control	53.2	382	53.6	349	−0.4	.347
High-expertise pro-author	61.3	321	66.5	327	−5.2	.001
Low-expertise pro-author	41.6	352	46.2	374	−4.6	.001

*Note:* *P*-values represent statistical difference in the gender gap. Full regression table from which predicted probabilities were derived can be found in Supplementary Material table B1.

When presented with no expertise cues, respondents select men and women information sources at a nearly equal rate (x^− ^= 0.4 percent). However, while both men and women pro-authors benefit from the introduction of high-expertise source cues, gains for women are sizably smaller, leading to a large gender gap. Respondents are 8.1 percent more likely to select a woman source in the high-expertise treatment relative to the control with no expertise cues. However, identical men gain in selection to a disproportionate degree from the high-expertise cue (12.9 percent), leading to a gender gap of 5.2 percent among equivalent men and women experts. This implies that the expertise of a woman information source is strongly discounted relative to an identical man, all else held equal.

Results indicate that the low-expertise treatment evinces the inverse pattern. Women are penalized for a lack of expertise more severely (11.6 percent decrease relative to the control) than their male counterparts (7.4 percent decrease relative to the control). This increases overall gender bias among equivalent low-expertise authors to 4.3 percent. This implies that low-expertise men are afforded a benefit of the doubt that is not afforded to women, even if their qualifications are equally lacking. Overall, this evidence suggests that male pundits have more to gain from expertise, and less to lose from a lack thereof, relative to equivalent female pundits.

Table 6 provides identical analyses to those presented previously while dividing the sample into feminine- and masculine-stereotyped issues. Overall, the increase in gender gap brought about by source expertise cues is consistent across both feminine- and masculine-stereotyped issues.

High (low) expertise cues result in roughly equal increases (decreases) relative to the control for both feminine and masculine issues. Across issues, women are rewarded less than men for increased levels of expertise and punished more severely for a lack of expertise. This includes issues that disproportionately affect women or where women would have more real-life experience, such as women’s health care or gender-based wage discrimination. This finding was also consistent based on respondent gender, with men and women in the sample exhibiting the same basic shifts in selection based on expertise and gender of the author (see Supplementary Material C). This is discouraging from a normative perspective, as audiences appear to trust women experts less than men even on vital issues where women’s self-interests should be highly salient.

**Table 6.** Gender bias based on expertise for feminine- and masculine-stereotyped issues.

	Feminine-stereotyped issue					
	Selects woman pro-author		Selects man pro-author		Gender gap	
	%	*n*	%	*n*	Gap, %	(*p*-value)
Control	57.7	197	58.0	164	−0.3	.368
High-expertise	65.8	164	58.0	188	−4.3	.001
Low-expertise	46.1	178	51.1	170	−5.0	.001

	Masculine-stereotyped issue					
	Selects woman pro-author		Selects man pro-author		Gender gap	
	%	*n*	%	*n*	Gap, %	(*p*-value)
Control	48.40	185	49.10	163	−0.70	.015
High-expertise	56.60	157	62.30	162	−5.70	.001
Low-expertise	36.90	174	42.00	204	−5.10	.001

*Note:* *P*-values represent statistical difference in the gender gap. Full regression table from which predicted probabilities were derived can be found in Supplementary Material table B1.

### Discussion

Results provide strong support for the hypotheses. When given no expertise cues, respondents find men to be more credible than women. Yet, this gender-based gap does not appear to affect news consumption habits on its own. After the introduction of expertise cues which make the pundits’ qualifications directly salient, individuals exhibit biased behavior that exacerbates the gender gap in selection. While women are rewarded for increasing levels of expertise, they gain far less relative to men. In addition, women are punished far more severely for a lack of expertise than identical men. This same pattern is consistent across masculine- and feminine-stereotyped issues and respondent gender. The normative implications of these results are troubling, as women may have to provide the audience with stronger credentials just to be viewed as equally qualified to men. The fact that this pattern remains consistent for feminine-stereotyped issues—and more specifically issues that more directly affect women—implies that women in the media often face an uphill battle to advocate for their interests.

While Study 1 demonstrates the challenges faced by women experts in the media, it tests the relationship between gender and expertise in a context that is absent of partisan cues. Thus, these results highlight gender expertise biases in a context where the partisanship of men and women pundits is less salient, such as when the pundits either lack or share a partisan identity. Yet it is less clear whether the expertise-based gender biases remain pertinent across party lines, and whether competing party cues and polarization moderate, overwhelm, or even exacerbate gender-based biases. I address this point in Study 2.

## Study 2

Study 1 provides strong evidence of gender biases regarding expertise in a context where partisanship is less salient, such as when a news source does not provide direct partisan cues or both pundits share a partisan identity or news network affiliation. Study 2 seeks to build upon Study 1 with the additional context of partisan cues to assess whether gender biases are mitigated or exacerbated by partisanship and polarization.

### Study Design

I leveraged a nationally representative sample of 804 respondents from Lucid, an online service (March 18–20, 2020). The design of Study 2 is similar to that of Study 1. Respondents viewed an identical set of headlines to those used in Study 1. Respondents viewed a total of 6 sets of headlines in a within-subjects design, bringing the effective sample size to 4,824 observations. However, unlike Study 1, I did not include a control condition featuring two men in Study 2, meaning that every set of headlines featured one man and one woman. I manipulated the expertise of the authors in an identical fashion to Study 1, resulting in three potential treatments: 1**)** a high-expertise pro-author versus a low-expertise con-author, 2**)** a low-expertise pro-author versus a high-expertise con-author, and 3**)** a control condition in which the expertise of the authors is held constant. For Study 2, I also manipulated the partisanship of the sources. I randomized the partisan affiliation of each author, with one writing for Fox News, the other for MSNBC, both of which tend to garner audiences that are nearly equal degrees to the right and left of the ideological center, respectively (Mitchell et al. 2014). These network-based partisan cues may be a less direct means of manipulating partisanship than traditional party cues, in which the researcher outright tells the audience whether each author is a Democrat or Republican. However, network-based partisan cues have been shown to be highly effective in triggering partisan and ideological considerations and nearly equal in strength to traditional party cues (Ozer and Wright 2022). Network-based cues have also been heavily utilized in prior selective exposure research as a manipulation of partisanship (Turner 2007; Iyengar and Hahn 2009; Ozer 2020). In addition, network-based partisan cues are a more realistic manipulation in a media context, as audiences are not explicitly informed about a pundit’s personal partisan identity unless that pundit worked directly for a party. The ultimate result is a 2 x 3 x 2 experiment (Woman pro-author/Man pro-author x High-expertise/Low-expertise/Control pro-author x In-party/Out-party pro-author).

### Measures

The main independent variables are the treatments assigned to the pro-author. I measure the expertise manipulation with binary indicators representing the expertise treatment: high-expertise pro-author, low-expertise pro-author, and control with no expertise cues. I use binary measures to indicate the assignment of the gender treatment: 1**)** woman pro-author versus man con-author, 2**)** woman pro-author versus man pro-author. Finally, I measure partisan congruence with the author by comparing the respondent’s partisanship (measured prior to treatment) to the partisan treatment. I used this to create a binary variable that captures partisan congruence (1 = In-party pro-author, 0 = Out-party pro-author).^7^ The main dependent variable is article selection, representing the respondent’s choice in news consumption. Respondents selected which of the two articles they would prefer to read if given the choice (1 = pro-author, 0 = con-author).^8^

### Results

First, to address the relative influence of source expertise, partisan congruence, and gender on news consumption, table 7 presents the coefficients from a binary logit regression featuring dichotomous indicators for the type of treatment, a fixed effect for issue type, and a random effect for the respondent (see Supplementary Material B, table B3 for full regression table).

**Table 7.** Main effects of selection based on source expertise, partisanship, and gender on news selection.

	Selection coefficient	
Treatment	(cluster-adjusted standard error)	*n*
Control	0.573	1,381
	(−0.002)	
High-expertise	0.64	1,453
	(−0.002)	
Low-expertise	0.495	1,390
	(−0.003)	
Man	0.572	2,164
	(−0.002)	
Woman	0.569	2,061
	(−0.002)	
In-party	0.64	2,099
	(−0.002)	
Out-party	0.502	2,125
	(−0.002)	

After accounting for partisanship and expertise, respondents select both men and women sources at roughly equivalent rates. Respondents instead appear to prioritize both partisanship and expertise over gender cues. Predicted probabilities reveal that respondents are 12.8 percent more likely to select an in-party source relative to an equivalent out-party source. In addition, respondents reward high-expertise sources (6.7 percent increase in selection) and punish low-expertise sources (7.8 percent decrease in selection) more often relative to the control. Overall, these results suggest that while individuals may hold biases based on gender roles, as seen in Study 1, deeply held partisan loyalties may often take precedent in terms of media choices.

To better account for treatment combinations, table 8 presents the coefficients from a binary logit regression featuring dichotomous indicators for each combination of treatment, fixed effects for the issue type, and a random effect for the respondent.

**Table 8.** Source expertise, partisanship, and gender and news selection.

Treatment	Selection Coefficient	
	(cluster-adjusted standard error)	*n*
Man out-party control	0.501	370
	(−0.003)	
Woman out-party control	0.481	322
	(−0.003)	
Man out-party high-expertise	0.578	342
	(−0.003)	
Woman out-party high-expertise	0.57	387
	(−0.003)	
Man out-party low-expertise	0.445	352
	(−0.003)	
Woman out-party low-expertise	0.43	328
	(−0.003)	
Man in-party control	0.66	353
	(−0.003)	
Woman in-party control	0.649	336
	(−0.003)	
Man in-party high-expertise	0.697	388
	(−0.003)	
Woman in-party high-expertise	0.718	336
	(−0.003)	
Man in-party low-expertise	0.541	358
	(−0.003)	
Woman in-party low-expertise	0.567	328
	(−0.003)	

Individuals once again display a consistent pattern of partisan bias, selecting in-party sources far more often than equivalent out-party sources. Results also provide support for the Expertise Hypothesis. High-expertise cues bolster selection of sources, while low-expertise cues undermine selection. This was true for both in-party and out-party sources, suggesting that while individuals may be predisposed to distrust the out-party, high levels of expertise may mitigate negative affective biases and resulting selective exposure to a notable degree.

Yet, while gender biases evince a strong interaction with expertise cues in Study 1, the addition of polarizing partisan cues appears to once again overshadow differences in selection between men and women experts. Table 8 shows that women in the out-party control condition are selected at nearly equal rates to men in the baseline out-party control condition. Moving down the table, while comparing men and women with identical levels of expertise and partisanship, results yield few differences based on gender. For example, high-expertise out-party men and high-expertise out-party women (the 3rd and 4th coefficients, respectively) are highly similar. This indicates that high-expertise cues increase selection of out-party pro-authors, and that this effect does not vary based on gender cues. Thus, while individuals may discount the perspective of women experts based on gender stereotypes, they also appear willing to overlook these potential violations of gender roles based on their partisan allegiances, at least in terms of selection.

### Discussion

Study 2 highlights the influence of partisanship and polarization in the political media sphere. Individuals are far more likely to select in-party sources relative to equivalent out-party sources. Results support the Expertise Hypothesis, however, as expertise cues help diminish these partisan-based gaps in selective exposure. Nonetheless, the introduction of partisan signaling appears to overwhelm the influence of gender cues. As a result, individuals appear to select men and women experts at nearly identical rates based primarily on the news network’s partisan congruence with the reader. In a sense, this means that news source selection becomes more egalitarian due to polarization: the audience is willing to overlook potential gender biases so long as a woman (man) is a member of the in- (out-)party. Yet, superseding gender stereotypes and biases with hyper-partisanship and polarization is likely not a positive normative outcome in terms of gender equality. The null effects of gender cues as well as their interaction with expertise do not necessarily imply that gender plays no role in individual acceptance of expert perspectives in the news. Indeed, Study 1 shows that gender roles play a rather substantial effect in the perception of experts across a range of issues under slightly less polarized circumstances.

However, findings from Study 2 nonetheless imply that when forced to choose between polarized in-party and out-party sources, individuals prioritize partisanship over gender.

This study is not without flaws. The subtlety of the gender cues—a simple name change for the author—may be undermining the effect of gender. This can be considered a strength in Study 1, showing that even very subtle gender cues trigger rather sizable gender biases against women experts. However, given the overwhelming strength of partisan cues, it is not unreasonable to expect that it would take far stronger signals of gender to produce comparable effects to those found in less blatantly polarized circumstances. As such, I address this in the conclusion as a potential avenue for further expansion.

## Conclusion

Across two studies, results show that biases against women in the news are exacerbated as expertise is made more salient to the discussion. While women experts do gain credibility and trust for increasing levels of expertise, these gains are substantially smaller than those of an identical man. Conversely, women are punished far more severely for a lack of expertise relative to identical men. In both cases, as expertise becomes more salient to the political discussion, the gap in perceived credibility between men and women grows. This is the case across a range of masculine- and feminine-stereotyped issues, including issues that have a disproportionate impact on women. This implies that women in political media face an uphill struggle, with women experts forced to appear more qualified in order to effectively communicate with the audience on important issues. However, this effect dissipates in a highly polarized context which pits members of opposing political parties against one another. In such instances, individuals prioritize partisanship (and expertise) over the gender of the in-/out-party source. Thus, while individuals may implicitly discount the perspective of an expert woman relative to an expert man, they appear willing to look past this if the woman expert is on their side of the proverbial partisan fence.

The normative implications of such results are mostly concerning. As a potential silver lining, these findings imply that women enjoy the same benefits of affective partisan polarization as men. And, more broadly, expertise cues shift individual news consumption in logical fashion irrespective of author gender while undermining polarization to a modest degree. However, the suggestion that individuals only value women experts to an equal degree when the alternative is a detested out-party source is far from what one would consider to be the pluralistic ideal. This brings with it the typical consequences of increased polarization and negative partisan biases that may prevent effective political communication and healthy democratic media consumption habits.

In addition, the consistency of results across masculine- and feminine-stereotyped issues supports prior works which suggest that direct gender cues and violations of gender roles activate gender biases to a greater degree than the issues themselves (Bauer 2020). Moreover, while women respondents appear to place more weight on expertise, gender biases are nonetheless prevalent. Ultimately, this suggests that while women’s representation in political media is important, representation alone may not be sufficient to counter gender biases. Instead, such biases are more deeply ingrained in societal norms and women’s expected congruence with these norms, necessitating a shift in societal views over time in order to mitigate.

Finally, while this research addresses the obstacles faced by women experts in political media, it leaves many questions unanswered, offering potential avenues for future research and expansion. A potential expansion upon this research may benefit from more explicit gender cues, perhaps including video of a television news panel akin to what one may see on major cable news networks. While such an approach would be cost intensive and require careful manipulation testing, research may yield insightful findings by manipulating combinations of the expertise and gender of various panel members. Additionally, the null effect of the baseline condition with no expertise cues raises questions regarding the credibility of men and women political pundits in scenarios where expertise is not made explicit on the screen. While reluctant to posit a post hoc hypothesis, I believe this unanswered question leaves ample opportunity for exploration via additional experimentation, the use of open-ended questions to uncover more nuanced trends in reasoning, and qualitative approaches that may help with theory building in this respect. In addition, future iterations of this research may benefit by expanding from a narrower assessment of gender and expertise to consider the interplay of other potential factors, including the news source’s potential conflicts of interest, race, and even perceptions of charisma or physical attractiveness.

## Supplementary Material

nfad011_Supplementary_Data

## Data Availability

Replication data and documentation are available at: https://osf.io/gd32b/?view_only=ac04bc81336448108e703fe0b1ec530a.

## Footnotes

1 Quoted in Anisman-Razin et al. (2018, p. 1).

2 Also see Coppock (2019) and Coppock and McClellan (2019) regarding other online survey sample services.

3 Respondents were exposed to the sets of headlines in random order.

4 Similar to approaches utilized by Anisman-Razin, Kark, and Saguy 2018; Ozer 2020.

5 Respondents selected which article they prefer, but were not made to read the article itself before moving on to the next set of headlines.

6 Difference-of-means testing yields substantively similar though less precise results.

7 “Pure independents” are omitted from analysis.

8 Respondents selected which article they prefer but were not made to read the article itself before moving on to the next set of headlines.
