# Supplementary material for: Women Experts and Gender Bias in Political Media
Source: Public Opin Q. 2023 May 11;87(2):293–315. doi: 10.1093/poq/nfad011 (PMC10408780; doi:10.1093/poq/nfad011)
Supplement: nfad011_Supplementary_Data [file nfad011_supplementary_data.docx]

SUPPLEMENTAL MATERIALS:

Women Experts and Gender Bias in Political Media

Adam L. Ozer

[oadam925@gmail.com](mailto:oadam925@gmail.com)

# TABLE OF CONTENTS

# A: Manipulation Examples

# B: Additional Analyses

# C: Analyses Based on Respondent Gender

# D: Manipulation Checks

# E: Results Based on Respondent Partisan Affiliation

# F: Balance Tables

# G: Full Survey Question Wording

# Supplementary Materials A: Manipulation Examples

## Study 1

Figure A1: Manipulation Example: Automatic Voter Registration


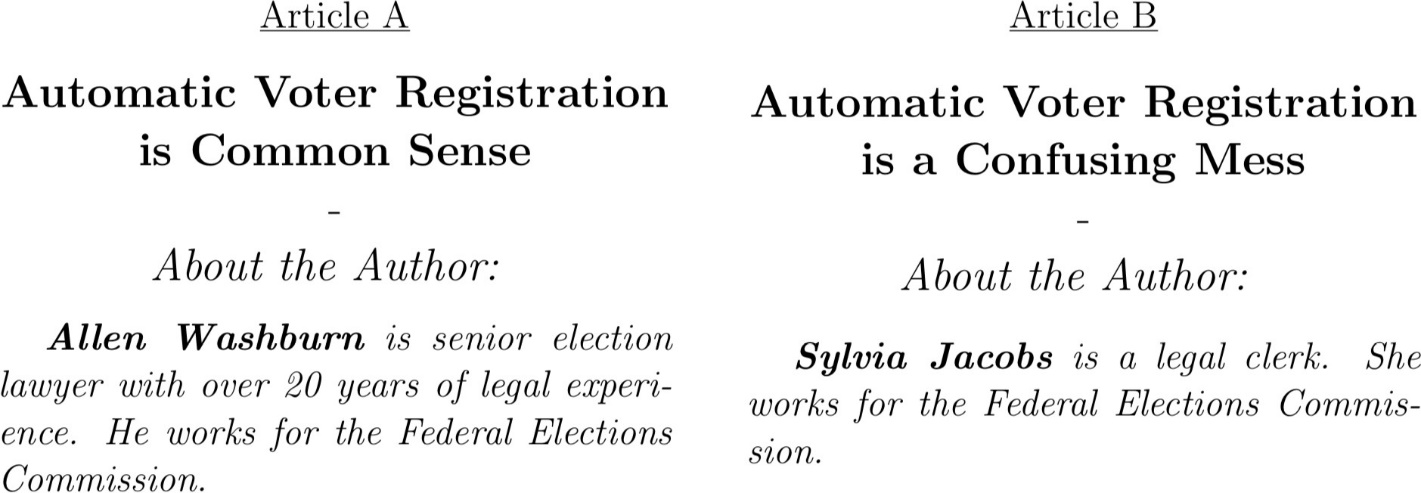


Figure A2: Manipulation Example: Birth Control


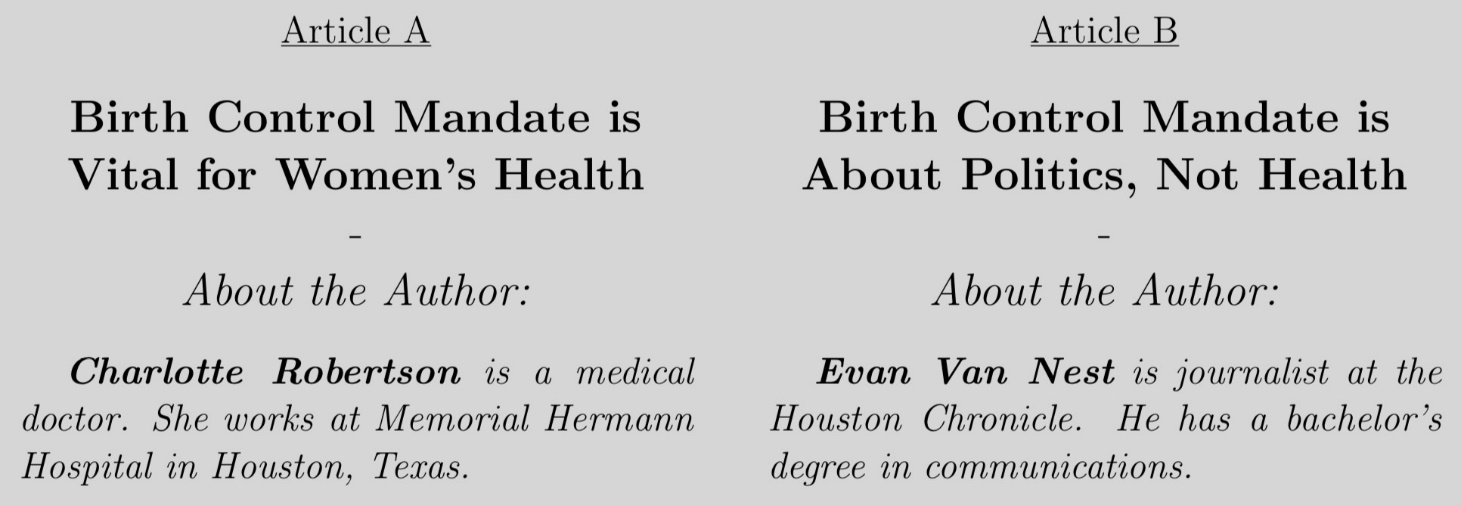


Figure A3: Manipulation Example: Drone Strikes


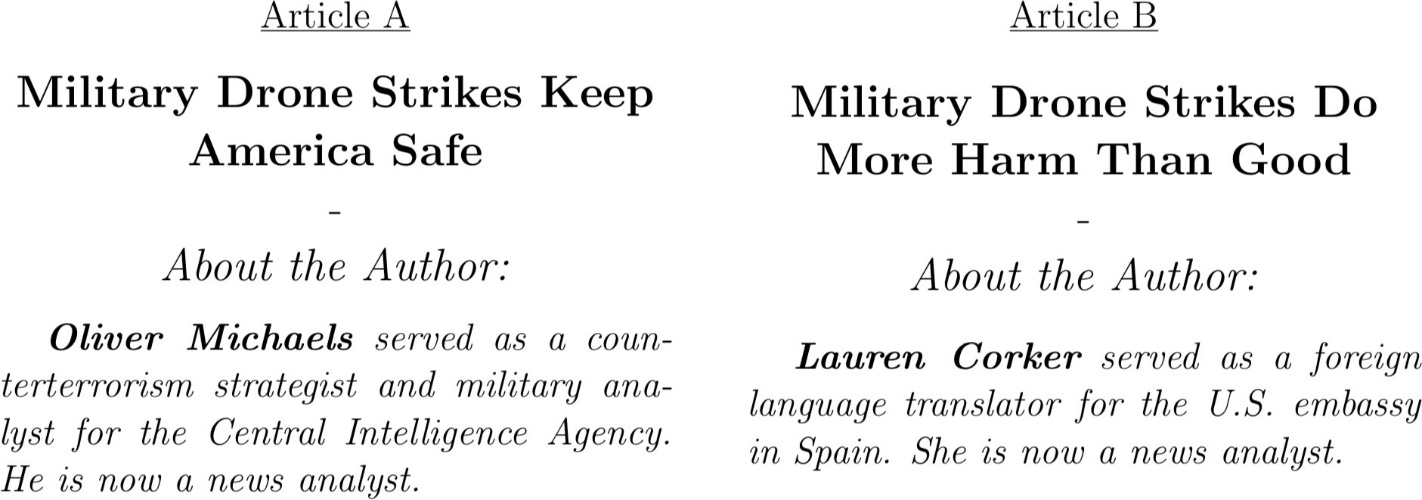


Figure A4: Manipulation Example: Gender Wage Gap


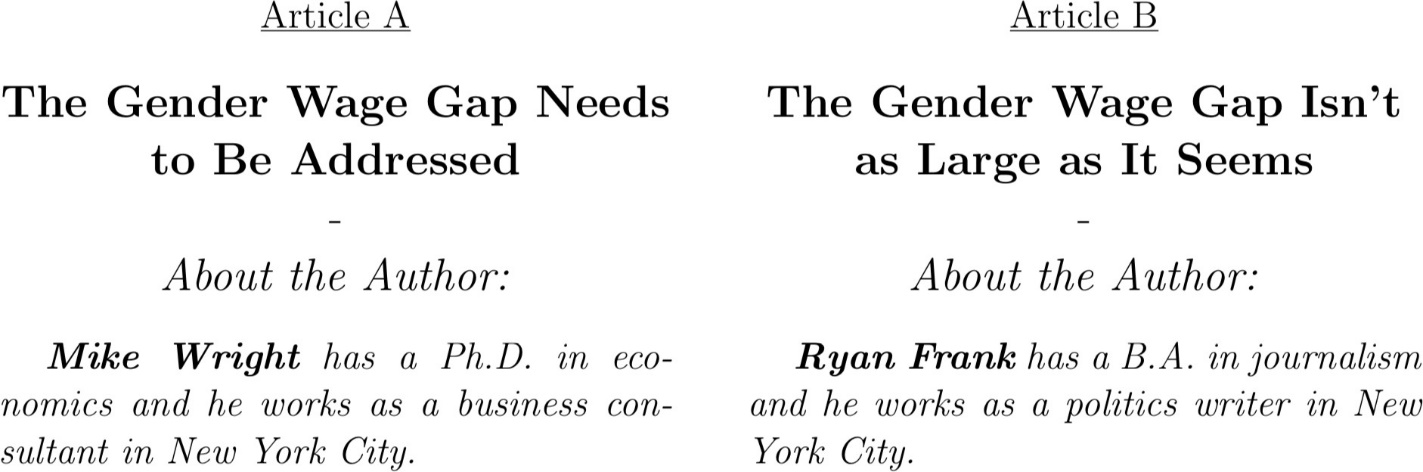


Figure A5: Manipulation Example: Paid Maternity Leave


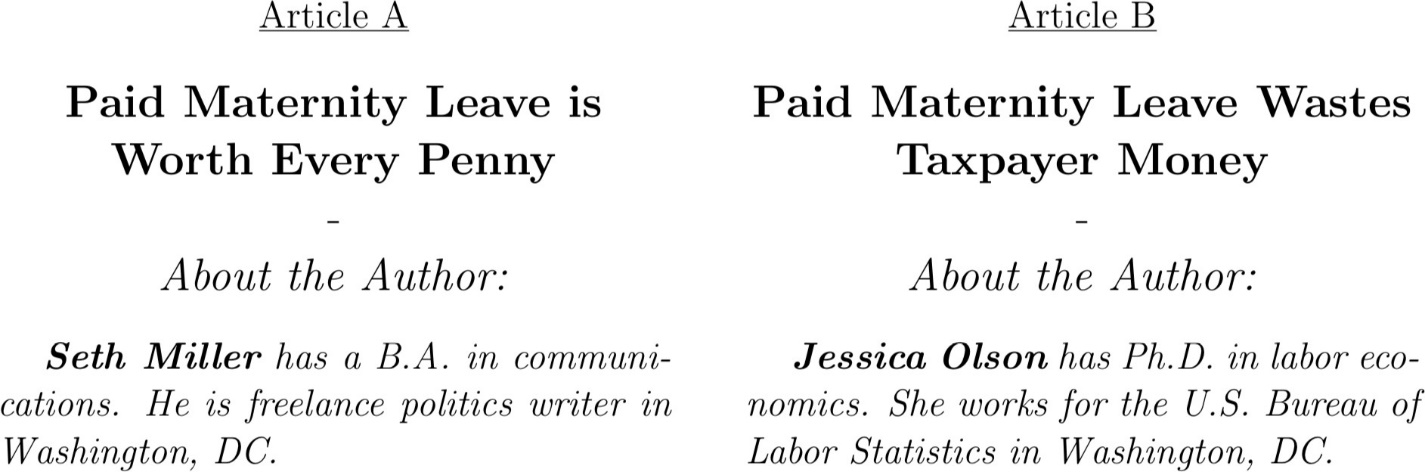


Figure A6: Manipulation Example: Trade Tariffs


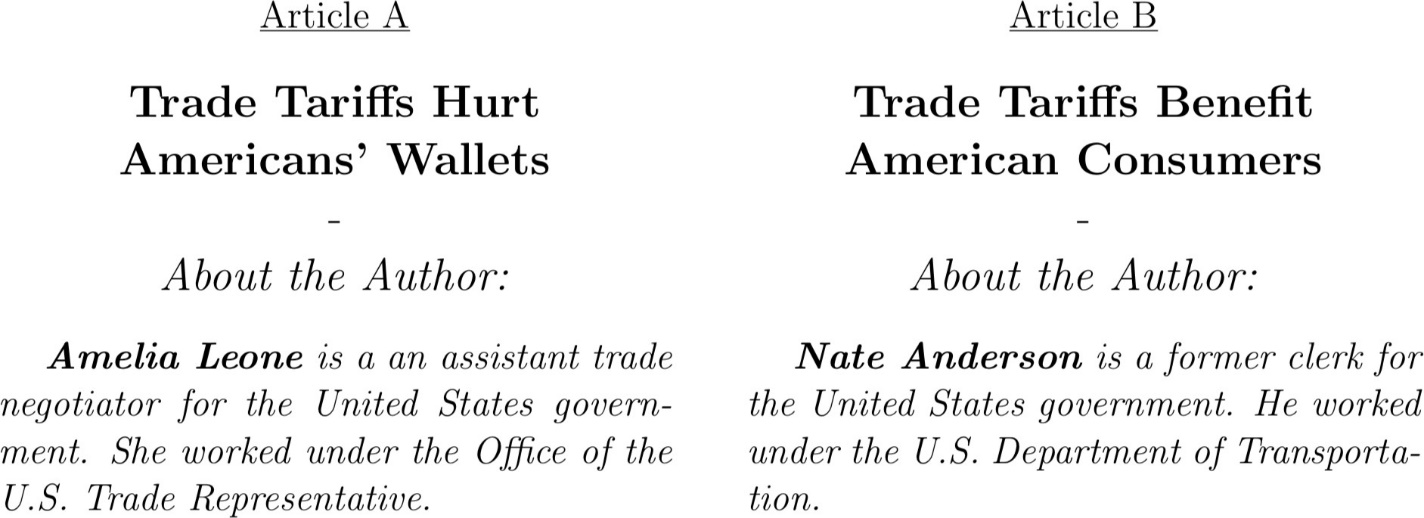


## Study 2

Figure A7: Manipulation Example: Automatic Voter Registration


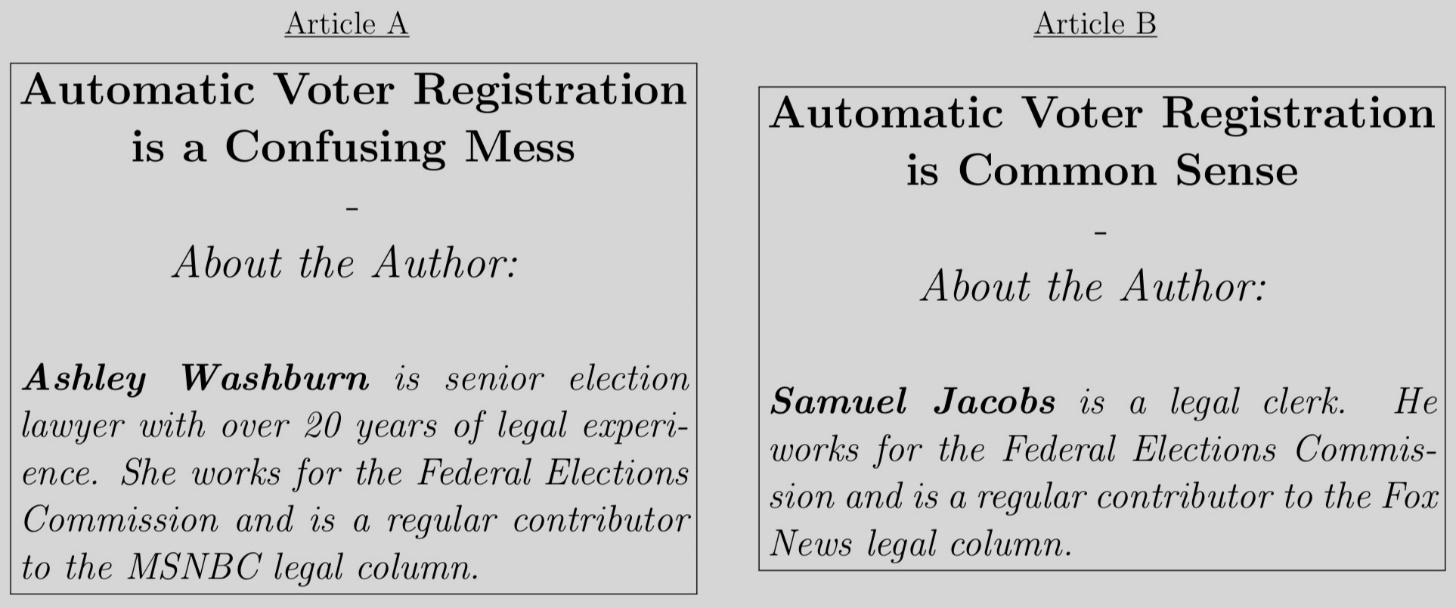


Figure A8: Manipulation Example: Birth Control


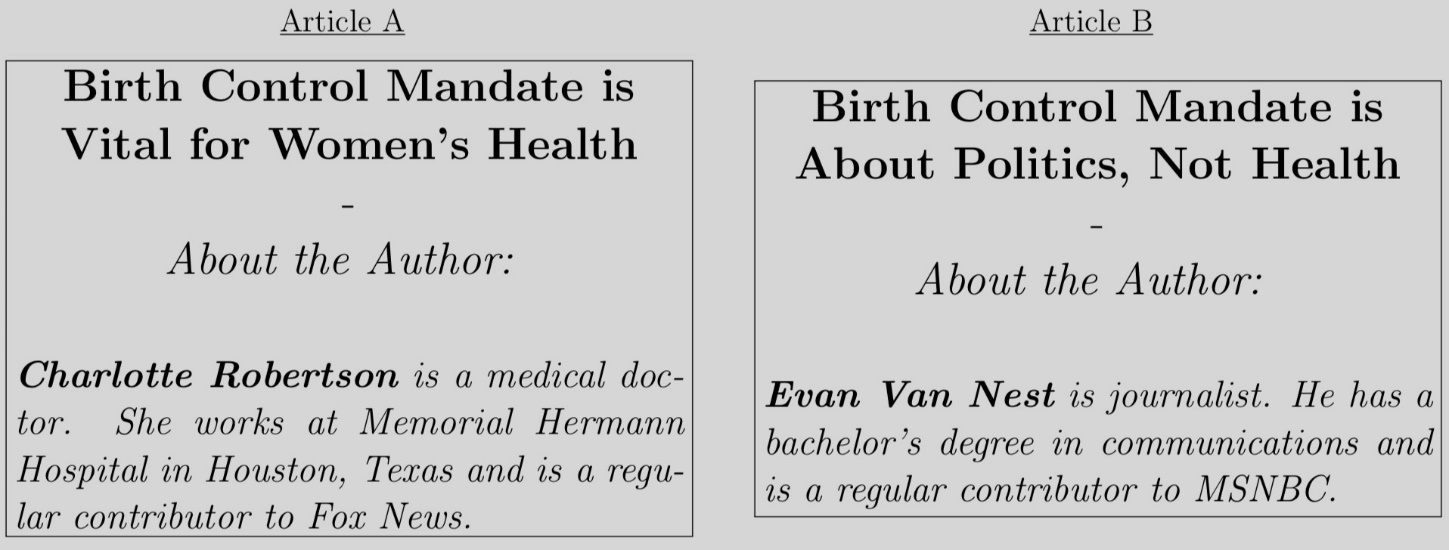


Figure A9: Manipulation Example: Drone Strikes


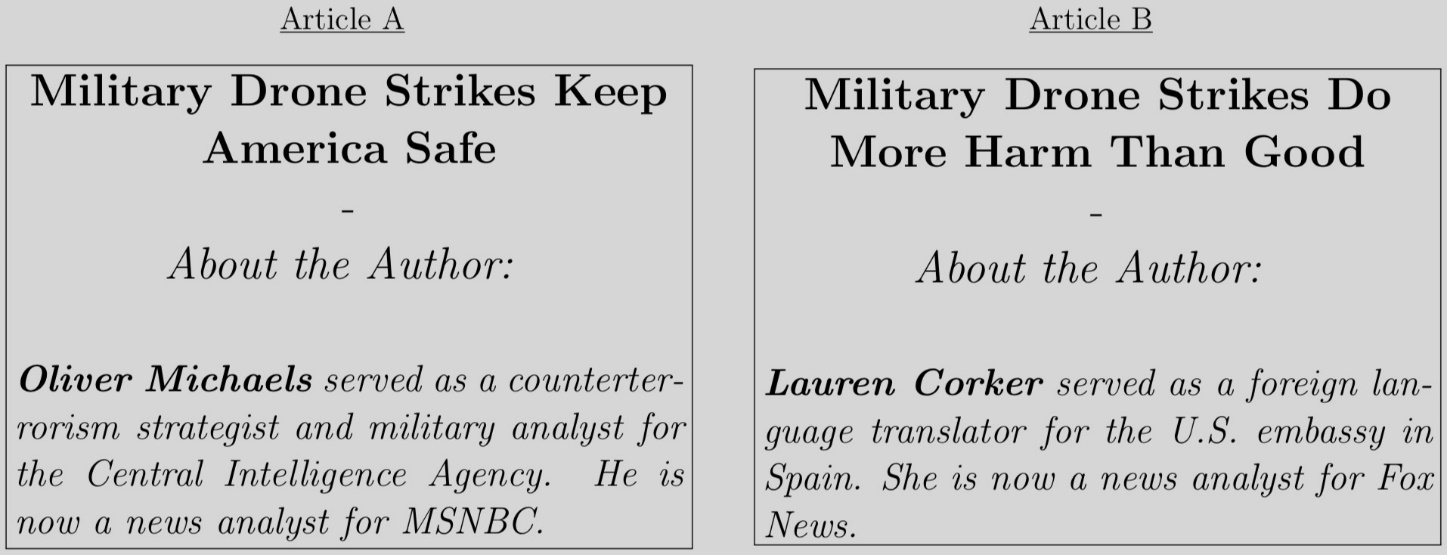


Figure A10: Manipulation Example: Gender Wage Gap


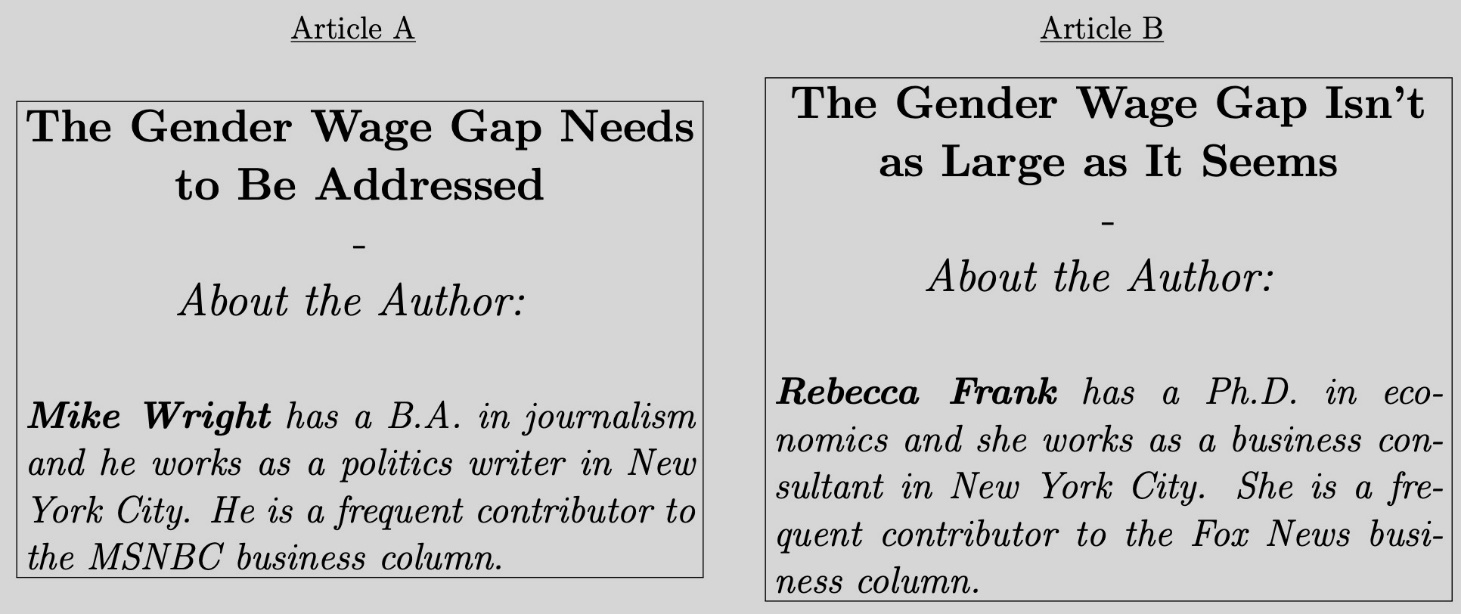


Figure A11: Manipulation Example: Paid Maternity Leave


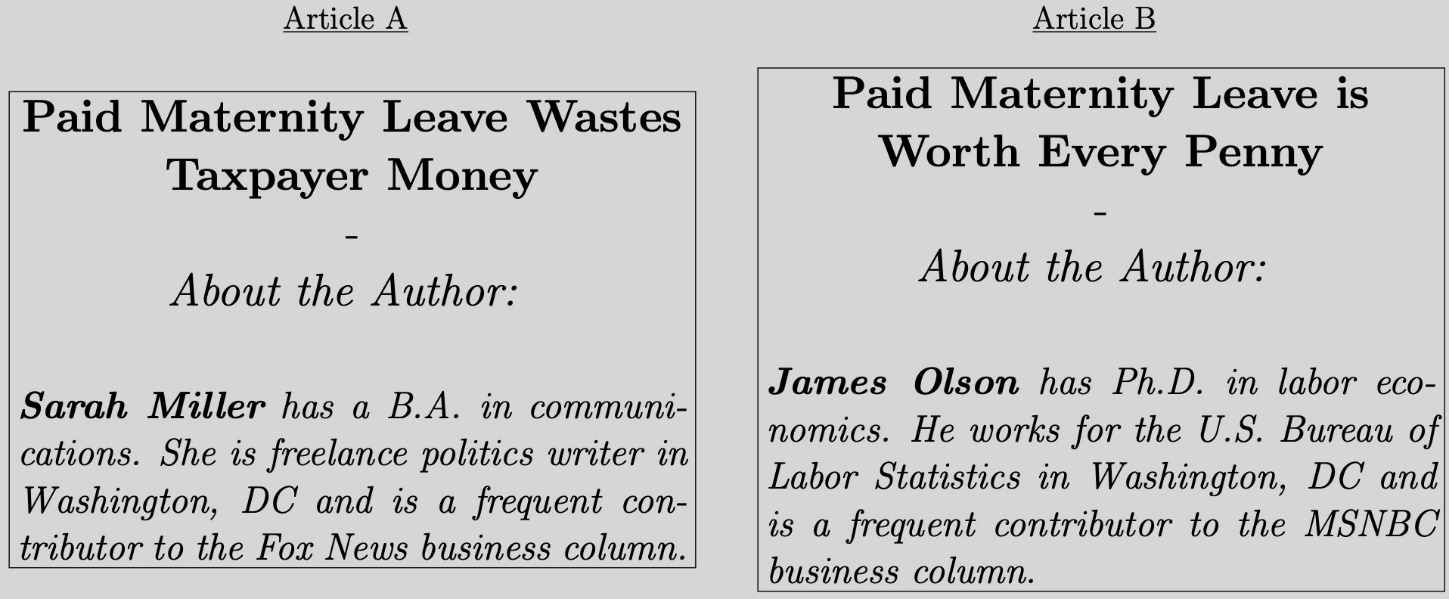


Figure A12: Manipulation Example: Trade Tariffs


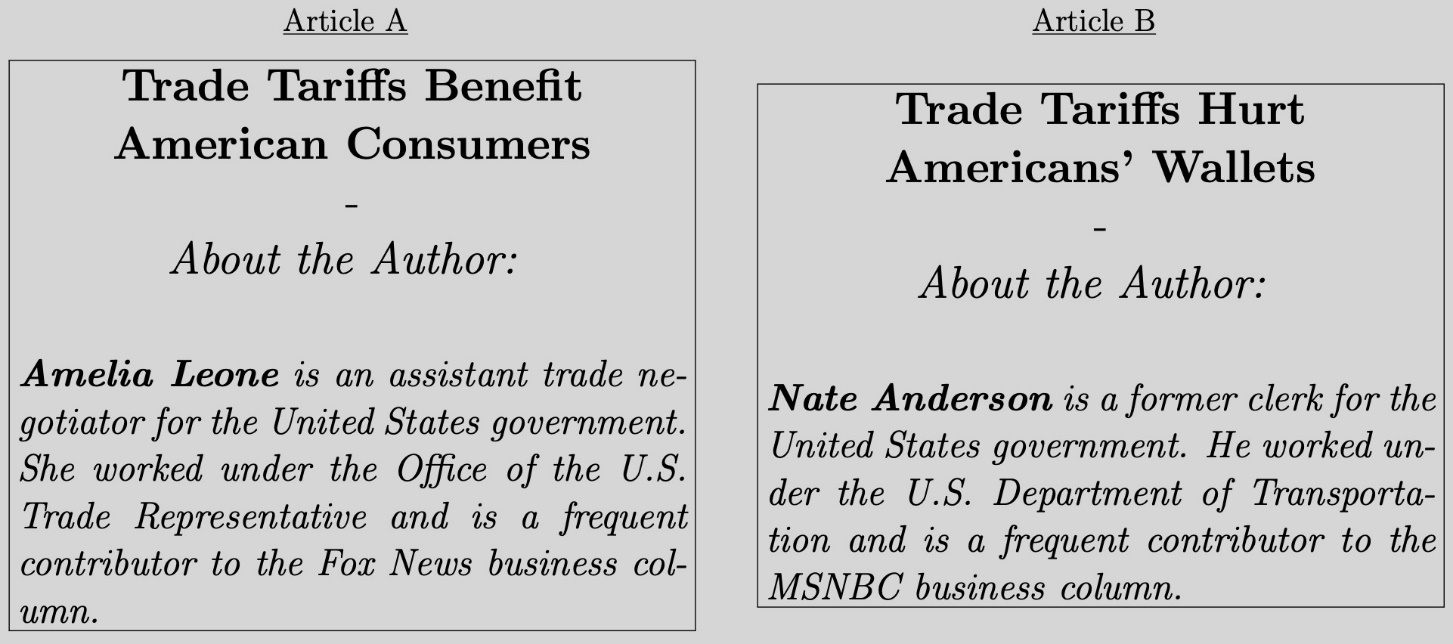


# Supplementary Materials B: Additional Analyses

## Study 1

Table B1 presents a logit regression analysis assessing the impact of expertise and gender cues on selection of the pro author article. Analysis utilizes binary indicators for the treatment

combinations, a binary indicator for whether the issue was feminine or masculine stereotyped, and a random effect for the respondent. Coefficients for Table B1 are illustrated in /textbfFigure M2 of the main text. Since raw logit regression output does not lend itself to easy substantive interpretations, please see the predicted probability tables found in the main text.

Table B1: News article selection based on author expertise and gender (n = 3,127)

| **Treatment** | **Selects the pro author article**  *Regression Coefficient*  *(S.E.; p-value)* |
| --- | --- |
| Man (no expertise cue) | -0.036  (0.163; p = .827) |
| Woman (no expertise cue) | -0.066  (0.157; p = .673) |
| High expertise man | 0.494  (0.164; p = .003) |
| High expertise control | 0.213  (0.161; p = .187) |
| High expertise woman | 0.285  (0.165; p = .085) |
| Low expertise man | -0.326  (0.158; p = .039) |
| Low expertise control | -0.419  (0.160; p = .001) |
| Low expertise woman | -0.520  (0.074: p = .009) |
| Feminine stereotyped issue | 0.383  (0.074; p = .001) |
| Constant | -0.004  (0.112; p = .971) |

Similar to Table B1, Table B2 presents a logit regression which assess the effect of the treatment combinations on the perception of pro author credibility relative to the con author. For the most part, results closely reflect those of found in the previous analysis regarding news consumption.

Table B2: Perceived source credibility based on author expertise and gender (n = 3,127)

| **Treatment** | **Says coauthor is more credible**  *Regression Coefficient*  *(S.E.; p-value)* |
| --- | --- |
| Man (no expertise cue) | -0.009  (0.162; p = .957) |
| Woman (no expertise cue) | -0.218  (0.156; p = .161) |
| High expertise man | 0.422  (0.162; p = .009) |
| High expertise control | 0.357  (0.161; p = .027) |
| High expertise woman | 0.261  (0.164; p = .112) |
| Low expertise man | -0.534  (0.158; p = .001) |
| Low expertise control | -0.615  (0.160; p = .001) |
| Low expertise woman | -0.971  (0.165; p = .001) |
| Feminine stereotyped issue | 0.181  (0.074; p = .015) |
| Constant | 0.038  (0.121; p = .756) |

## Study 2

Table B3 presents a logit regression assessing the main effects of expertise, partisanship, and gender on selection of the pro author headline This analysis features binary indicators for each type of treatment cue, collapsing across combinations to isolate each type of cue. As a result, this analysis does not take into account the effect of interactions between different combination of treatments. Once again, analysis features a fixed effect for feminine and masculine issues stereotypes and a random effect for the respondent. Coefficients from this analysis are presented in Table 7 of the main text, with predicted probabilities discussed within the related text. While raw logit regression output does not lend itself easily to interpretation, results from Table B3 indicate that the high expertise, low expertise cues, and partisan cues shifted news consumption in the anticipated manner. The effects of these cues appear to overwhelm that of gender cues.

Table B3: Main effect of source expertise, partisanship, and gender on news consumption (n = 4,221)

| **Treatment** | **Selects the pro author**  *Regression coefficient*  *(Standard error; p-value)* |
| --- | --- |
| High expertise pro author | 0.290  (0.080; p = .001) |
| Low expertise pro author | -0.308  (0.079; p = .001) |
| Woman pro author | 0.006  (0.065; p = .932) |
| In-party pro author | 0.584  (0.065; p = .001) |
| Feminine stereotyped issue | 0.414  (0.065; p = .001) |
| Constant | -0.200  (0.080; p = .016) |

# Supplementary Materials C: Analyses Based on Respondent Gender

Table C1 presents predicted probabilities from Study 1 based on the respondents’ reported sex. Results show few if any differences between male and female respondents, suggesting that members of both sexes are roughly equally prone to the gender biases that are exacerbated by source expertise.

Table C1: Selection of the pro author based on respondent sex and experimental treatment

|  | **Selects woman pro author (Women respondents)**  Percentage  [n; number of tasks] | **Selects man pro author (Women respondents)**  Percentage  [n; number of tasks] | **Gender gap (Women respondents)**  Percentage  (p-value) | **Selects woman pro author (Men respondents)**  Percentage  [n; number of tasks] | **Selects man pro author (Men respondents)**  Percentage  [n; number of tasks] | **Gender gap (Men respondents)**  Percentage  (p-value) |
| --- | --- | --- | --- | --- | --- | --- |
| **Control** | 53.5%  [n = 204; 6] | 53.9%  [n = 173; 6] | **-0.7%**  **(p = .085)** | 52.8%  [n = 178; 6] | 53.8%  [n = 154; 6] | **-0.3%**  **(p = .623)** |
| **High expertise** | 61.4%  [n = 173; 6] | 66.9%  [n = 178; 6] | **-5.3%**  **(p = .001)** | 61.1%  [n = 148; 6] | 66.1%  [n = 171; 6] | **-5.0%**  **(p = .001)** |
| **Low expertise** | 41.9%  [n = 175; 6] | 46.7%  [n = 204; 6] | **-4.8%**  **(p = .001)** | 41.3%  [n = 177; 6] | 45.6%  [n = 170; 6] | **-4.3%**  **(p = .001)** |

Note: two-tailed t-test. P-values represent statistically significant difference in the gender gap.

# Supplementary Materials D: Manipulation Checks

Table D1 presents a manipulation check assessing the whether the expertise and gender manipulations affected respondents’ perceptions of perceived ideology. Results indicate that the manipulations had little impact on perceptions of the author’s ideology. This is encouraging, as it allays concerns regarding potential confounds driven by the perception that the high expertise author or woman author was too liberal. However, the automatic voter registration issue frame exhibited some unanticipated effects. High expertise pro authors in the automatic voter registration manipulation are perceived to be slightly more conservative rather than more liberal. In addition, men arguing in favor of automatic voter registration are perceived to be slightly more conservative. While unanticipated and counterintuinve, this does raise a potential concern regarding confounding. However, this does not appear to effect the substantive results for the automatic voter registration frame, which closely match those of the other issues (see Supplementary Materials C). It is also possible that these effects are due to a multiple comparisons issue.

Table D1: Effect of experimental manipulations on perceived ideology of the pro author

*Dependent variable:*

Perceived ideology (Likert scale; 1 (Extremely liberal) - 7 (Extremely conservative)

|  | **Paid family leave**  *Coefficient*  *(Standard error)*  *p-value* | **Wage gap** | **Birth control** | **Automatic voter registration** | **Tariffs** | **Drone strikes** |
| --- | --- | --- | --- | --- | --- | --- |
| High expertise pro author | 0.198 | 0.018 | *−*0.046 | 0.512 | 0.070 | 0.059 |
|  | (0.175)  p = .257 | (0.160)  p = .913 | (0.163)  p = .776 | (0.177)  p = .004 | (0.155)  p = .650 | (0.162)  p = .715 |
| Low expertise pro author | *−*0.043 | 0.252 | *−*0.188 | 0.105 | *−*0.185 | *−*0.039 |
|  | (0.168)  p = .798 | (0.165)  p = .127 | (0.167)  p = .263 | (0.157)  p = .506 | (0.153)  p = .227 | (0.152)  p = .798 |
| Woman pro author | 0.134 | 0.083 | *−*0.083 | 0.067 | *−*0.009 | *−*0.408 |
|  | (0.162)  p = .409 | (0.173)  p = .631 | (0.167)  p = .622 | (0.171)  p = .694 | (0.154)  p = .952 | (0.160)  p = .011 |
| Man pro author | 0.132 | 0.036 | 0.157 | 0.434 | 0.083 | *−*0.017 |
|  | (0.180)  p = .464 | (0.158)  p = .818 | (0.163)  p = .336 | (0.162)  p = .008 | (0.151)  p = .583 | (0.158)  p = .916 |
| Constant | 3.363 | 3.323 | 3.642 | 3.375 | 4.195 | 4.315 |
|  | (0.161)  p = .001 | (0.152)  p = .001 | (0.156)  p = .001 | (0.146)  p = .001 | (0.139)  p = .001 | (0.146)  p = .001 |
| Adjusted R^2^ | *−*0.001 | *−*0.002 | *−*0.001 | 0.031 | *−*0.001 | *−*0.001 |

Table D2 presents a manipulation check assessing the whether the expertise and gender manipulations affected respondents’ perceptions of perceived expertise. I asked respondents how well the following terms described each author: Knowledgeable, experienced, qualified, and competent. Respondents rated authors on five-point Likert scales (1 = “Not well at all”, 5 = ”Extremely well”). I averaged the answers into one reliable scale of perceived expertise (Cronbach’s *α* = .92) and rescaled the index to run from 0 to 1 for ease of interpretation. Should the manipulations work as intended, one would expect a positive coefficient for the high expertise pro author treatment and a negative coefficient for the low expertise pro author treatment (both relative to the control with no expertise cues).

Results indicate that the manipulations are successful across issues. Overall, high expertise treatment increased perceptions of author expertise by 9.1% while low expertise cues decreased perceived expertise by 8.6%. The phenomenon was consistent across issue frames, suggesting that each individual cue was successful in manipulating the intended perceptions in the anticipated direction.

Table D2: Effect of experimental manipulations on perceived expertise of the pro author

*Dependent variable:*

Perceived expertise (Continuous scale; 0 = Lowest expertise, 1 = Highest expertise)

|  | **All issues**  *Coefficients*  *(Std. error)*  *p-value* | **Automatic voter registration** | **Tariffs** | **Drone strikes** | **Paid family leave** | **Wage gap** | **Birth control** |
| --- | --- | --- | --- | --- | --- | --- | --- |
| High expertise pro author | 0.091 | 0.098 | 0.064 | 0.047 | 0.021 | 0.127 | 0.064 |
|  | (0.011)  p = .001 | (0.024)  p = .001 | (0.020)  p = .002 | (0.021)  p = .022 | (0.034)  p = .532 | (0.022)  p = .001 | (0.022)  p = .004 |
| Low expertise pro author | *−*0.086 | *−*0.074 | *−*0.060 | *−*0.081 | *−*0.101 | *−*0.043 | *−*0.102 |
|  | (0.011)  p = .001 | (0.021)  p = .001 | (0.020)  p = .001 | (0.019)  p = .001 | (0.032)  p = .002 | (0.022)  p = .053 | (0.023)  p = .001 |
| Woman pro author | 0.012 | 0.014 | 0.013 | *−*0.0001 | 0.066 | *−*0.008 | 0.018 |
|  | (0.011)  p = .303 | (0.023)  p = .533 | (0.020)  p = .522 | (0.020)  p = .996 | (0.031)  p = .034 | (0.023)  p = .720 | (0.023)  p = .426 |
| Man pro author | *−*0.011 | *−*0.008 | 0.027 | 0.034 | *−*0.030 | *−*0.021 | *−*0.042 |
|  | (0.011)  p = .314 | (0.021)  p = .699 | (0.020)  p = .176 | (0.020)  p = .090 | (0.034)  p = .376 | (0.021)  p = .315 | (0.022)  p = .057 |
| Constant | 0.519 | 0.612 | 0.590 | 0.602 | 0.612*^∗∗^* | 0.585 | 0.634 |
|  | (0.010)  p = .001 | (0.019)  p = .001 | (0.018)  p = .001 | (0.019)  p = .001 | (0.028)  p = .001 | (0.021)  p = .001 | (0.021)  p = .001 |
| Adjusted R^2^ | 0.078 | 0.100 | 0.065 | 0.072 | 0.056 | 0.108 | 0.101 |

# Supplementary Materials E: Results based on respondent partisan affiliation

Table E1 presents the coefficients regression results similar to those of Table B1 assessing

whether or not the respondent chose the pro author based on the experimental treatment. However, the sample has been divided into Democratic and Republican respondents. Partisan leaners (i.e. Indepedents that reported leaning Republican/Democrat on the traditional

seven-point Likert scale) were included in the party towards which they lean. Pure independents were not included in this anaylsis. Results from this analysis yield few major substantive or statistically distinguishable diferences in selection based on expertise or gender between Democrats and Republicans.

Figure E1: Results based on respondent partisanship


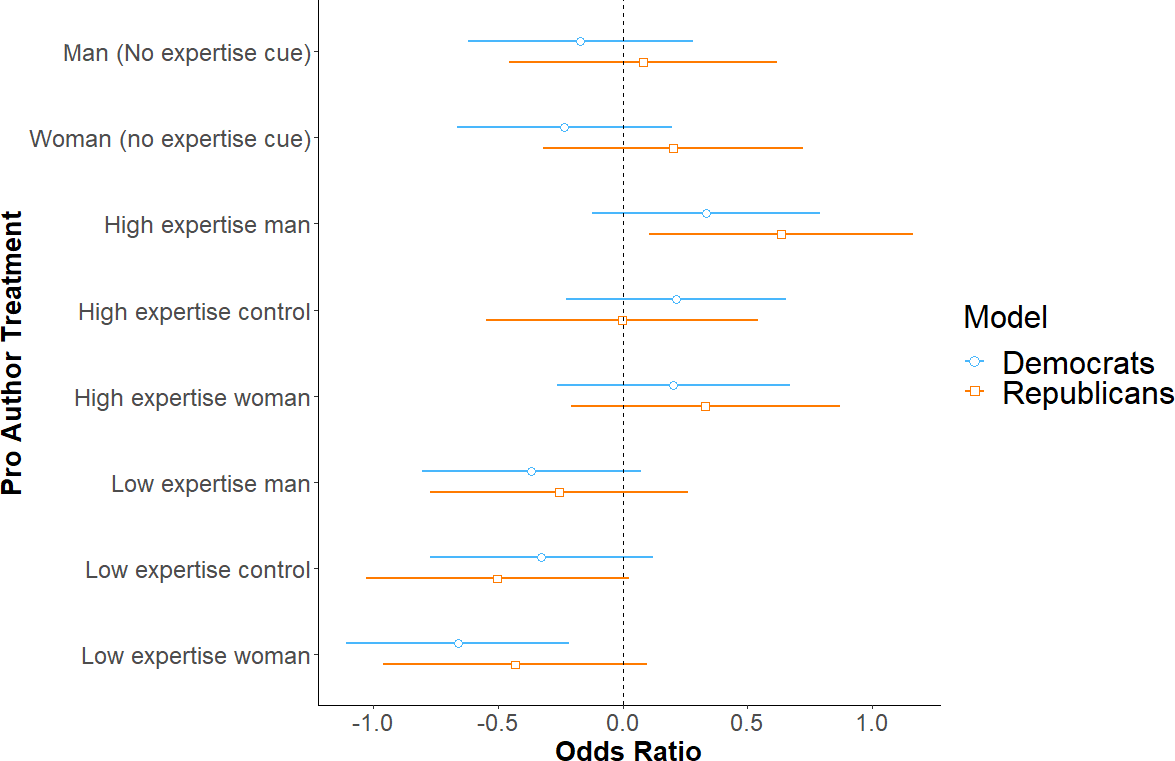


# Supplementary Materials F: Balance Tables

Tables F1 and F2 present the mean scores for demographic variables for Studies 1 and 2 respectively. Subsequent statistical testing reveals few substantive or statistically significant differences in demographic balance across treatments.

- Party ID: 7-point ordinal Likert scale; 1 = Strong Democrat, 7 = Strong Republican.
- Female: 1 = Female respondent, 0 = Male respondent.
- Age: Continuous (18+).
- Education: 8-point ordinal scale; 1 = ¡ High school degree, 8 = Doctoral Degree or equivalent.
- Income: 24-point ordinal scale; 1 = $14,000, 24 = ? $250,000.

Table F1: Balance Table (Study 1)

| Treatment | N | Party ID | Female | Age | White | Education | Income |
| --- | --- | --- | --- | --- | --- | --- | --- |
| 1 Control (no expertise cue) | 315 | 3.55 | 0.51 | 46.72 | 0.72 | 4.29 | 6.97 |
| 2 Man (no expertise cue) | 327 | 3.69 | 0.52 | 45.61 | 0.76 | 4.56 | 8.57 |
| 3 Woman (no expertise cue) | 382 | 3.61 | 0.53 | 46.34 | 0.76 | 4.40 | 7.59 |
| 4 High expertise man | 349 | 3.85 | 0.50 | 45.70 | 0.73 | 4.20 | 7.39 |
| 5 High expertise control | 348 | 3.52 | 0.55 | 44.83 | 0.73 | 4.33 | 7.74 |
| 6 High expertise woman | 321 | 3.80 | 0.54 | 46.25 | 0.72 | 4.28 | 7.63 |
| 7 Low expertise man | 374 | 3.73 | 0.54 | 46.77 | 0.75 | 4.54 | 7.91 |
| 8 Low expertise control | 359 | 3.77 | 0.53 | 45.47 | 0.75 | 4.32 | 7.80 |
| 9 Low expertise woman | 352 | 3.77 | 0.50 | 46.13 | 0.77 | 4.40 | 8.10 |

Table F2: Balance Table (Study 2)

| Treatment | N | Party ID | Female | White | Education | Income |
| --- | --- | --- | --- | --- | --- | --- |
| 1 Out-party man (no expertise cue) | 370 | 3.58 | 0.54 | 0.74 | 4.48 | 9.02 |
| 2 In-party man (no expertise cue) | 353 | 3.64 | 0.51 | 0.77 | 4.42 | 8.73 |
| 3 In-party woman (no expertise cue) | 336 | 3.77 | 0.54 | 0.72 | 4.38 | 8.74 |
| 4 Out-party woman (no expertise cue) | 322 | 3.69 | 0.54 | 0.77 | 4.50 | 9.26 |
| 5 High expertise in-party man | 388 | 3.76 | 0.50 | 0.80 | 4.63 | 9.50 |
| 6 High expertise in-party woman | 336 | 3.48 | 0.50 | 0.73 | 4.56 | 9.48 |
| 7 High expertise out-party man | 342 | 3.57 | 0.48 | 0.74 | 4.63 | 8.99 |
| 8 High expertise out-party woman | 387 | 3.62 | 0.54 | 0.74 | 4.51 | 9.03 |
| 9 Low expertise in-party man | 358 | 3.53 | 0.52 | 0.77 | 4.42 | 9.03 |
| 10 Low expertise in-party woman | 328 | 3.70 | 0.53 | 0.73 | 4.41 | 8.68 |
| 11 Low expertise out-party man | 352 | 3.72 | 0.51 | 0.73 | 4.64 | 9.55 |
| 12 Low expertise out-party woman | 352 | 3.55 | 0.51 | 0.72 | 4.40 | 9.04 |

**Supplementary Materials G: Full Question Wording**

**
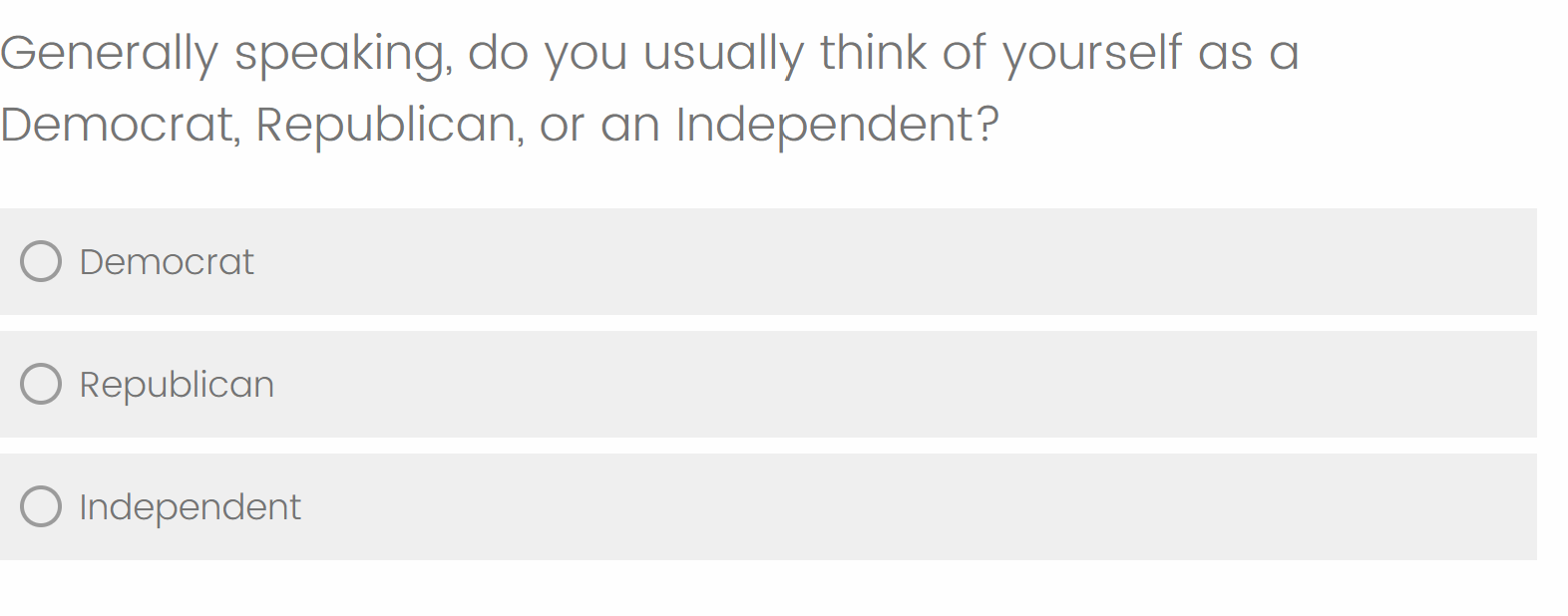
**

**
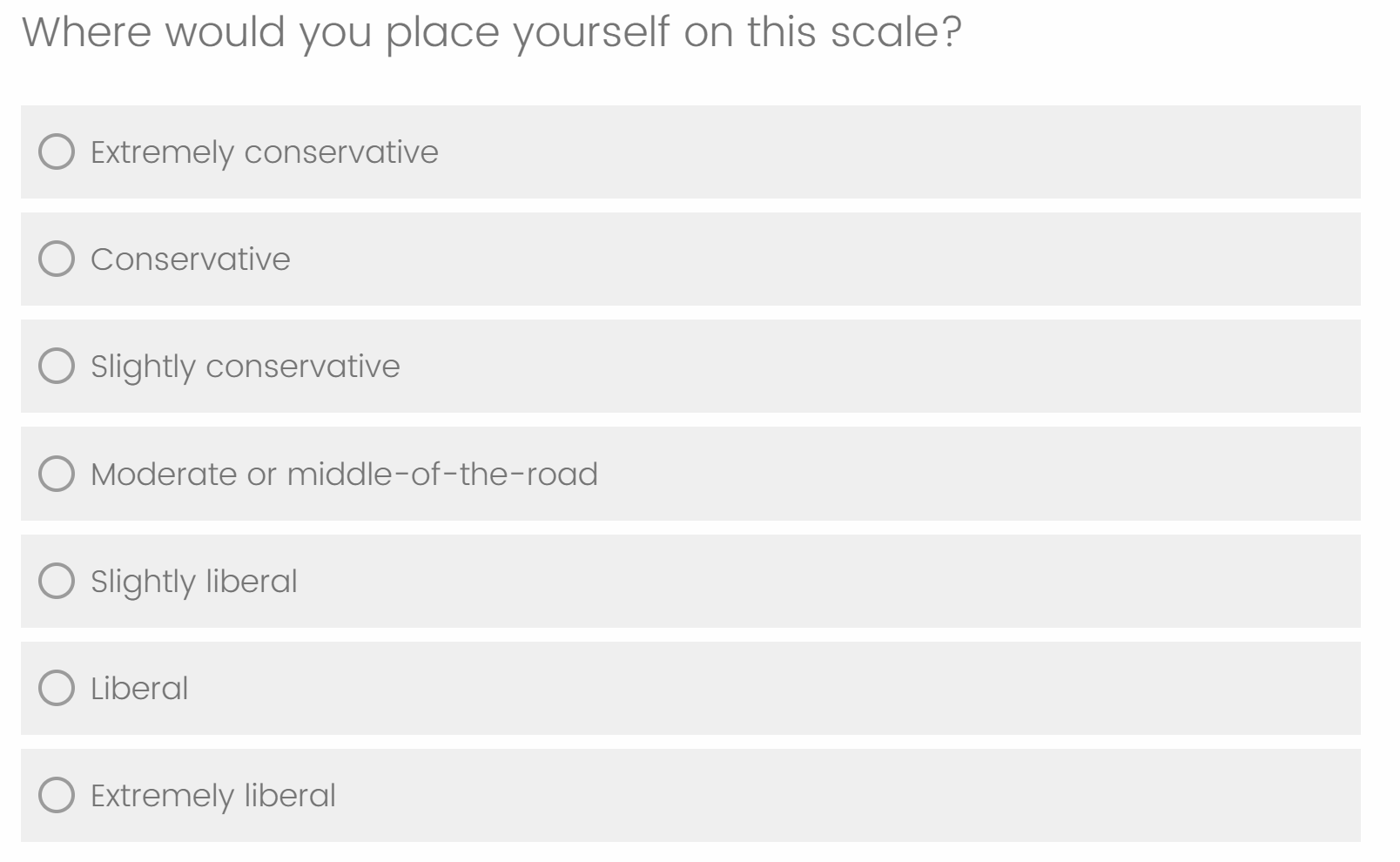
**

**
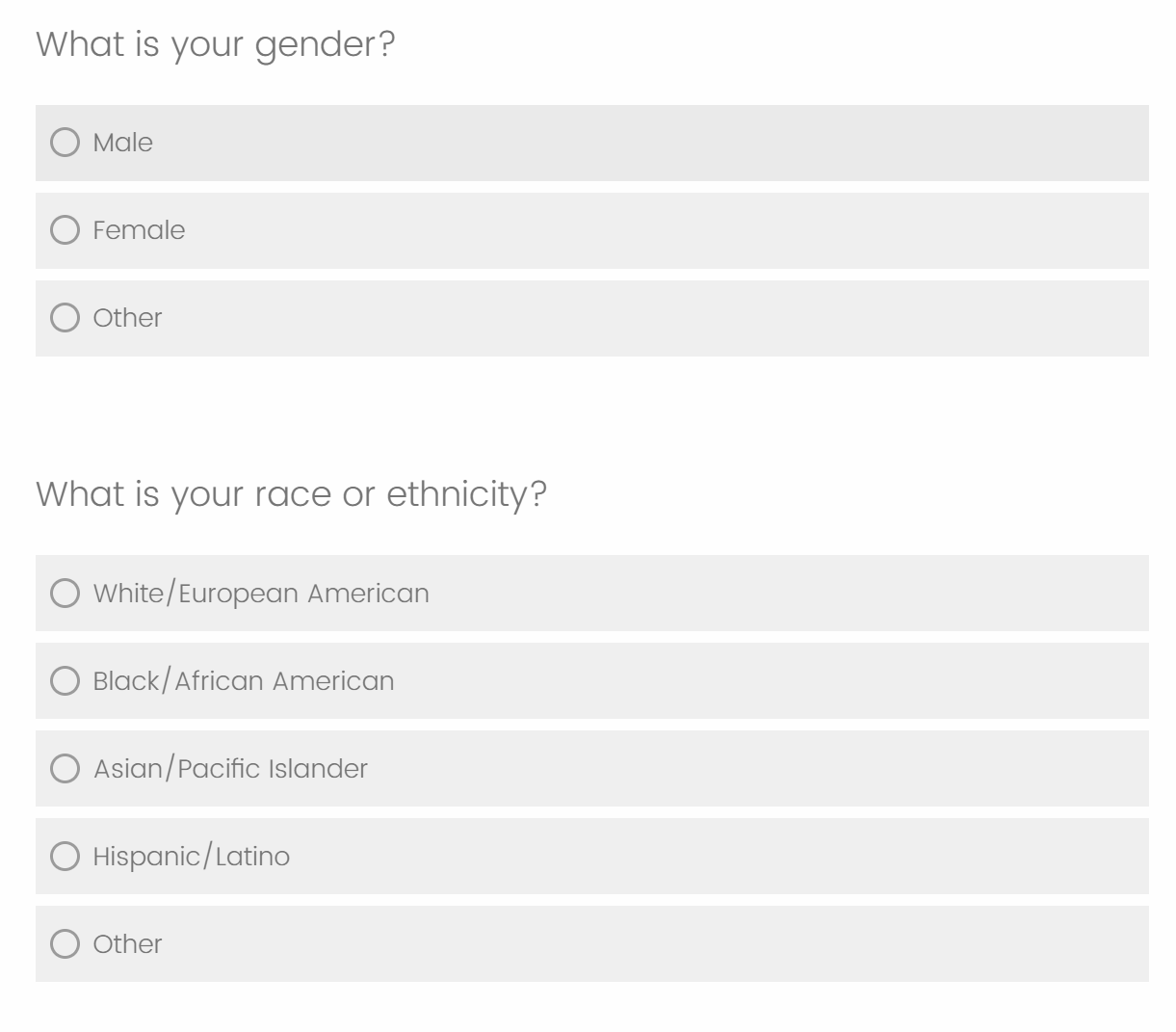
**

**
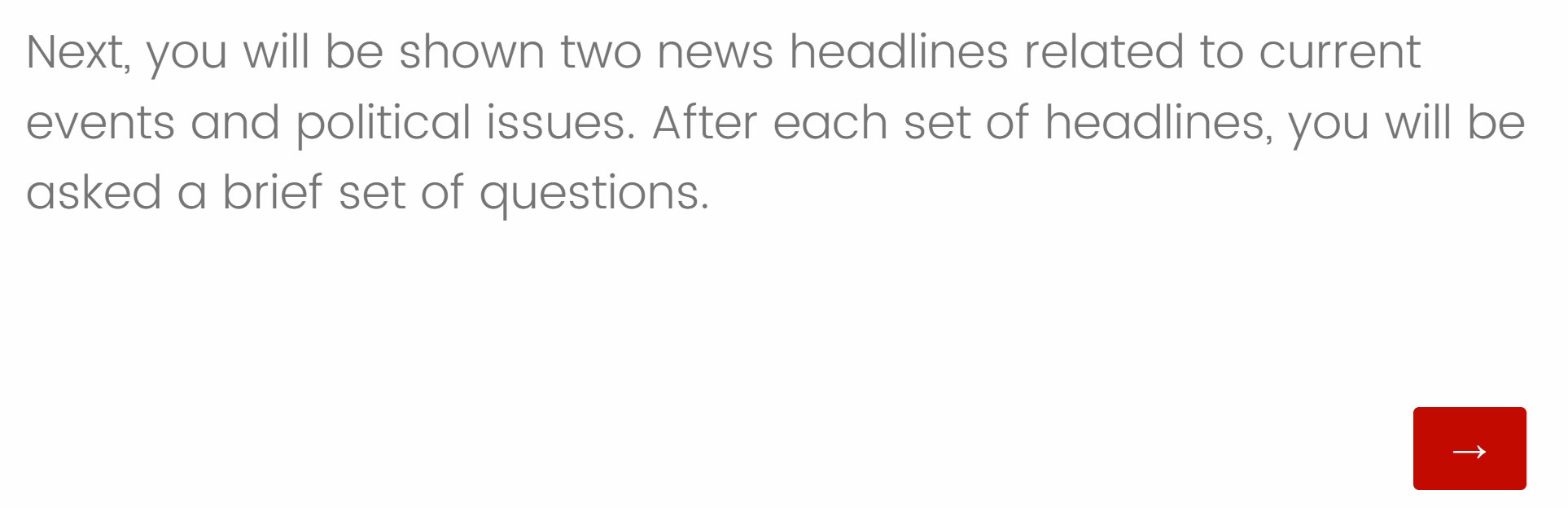
**

**< MANIPULATION HERE >**

**
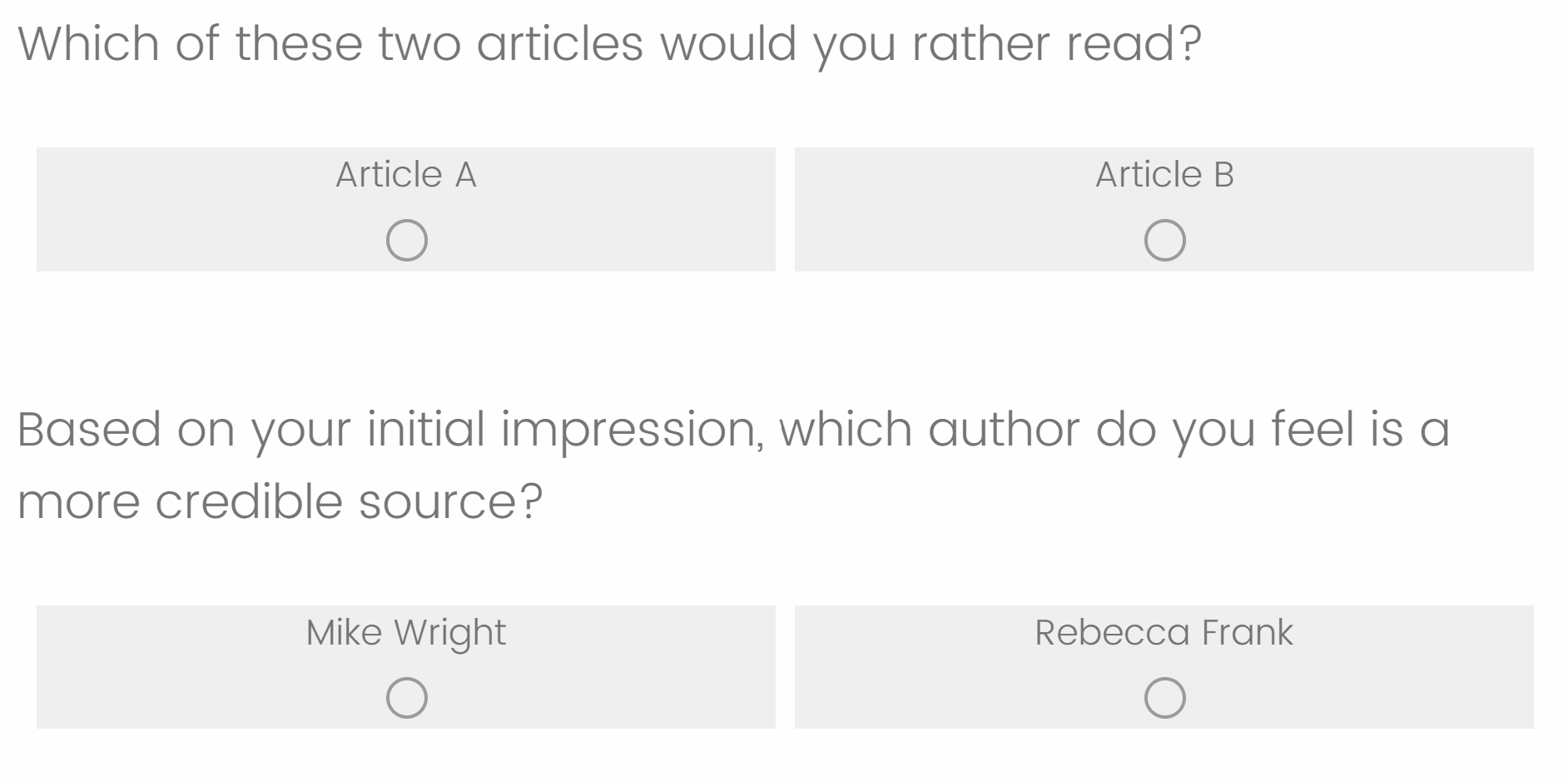
**

**
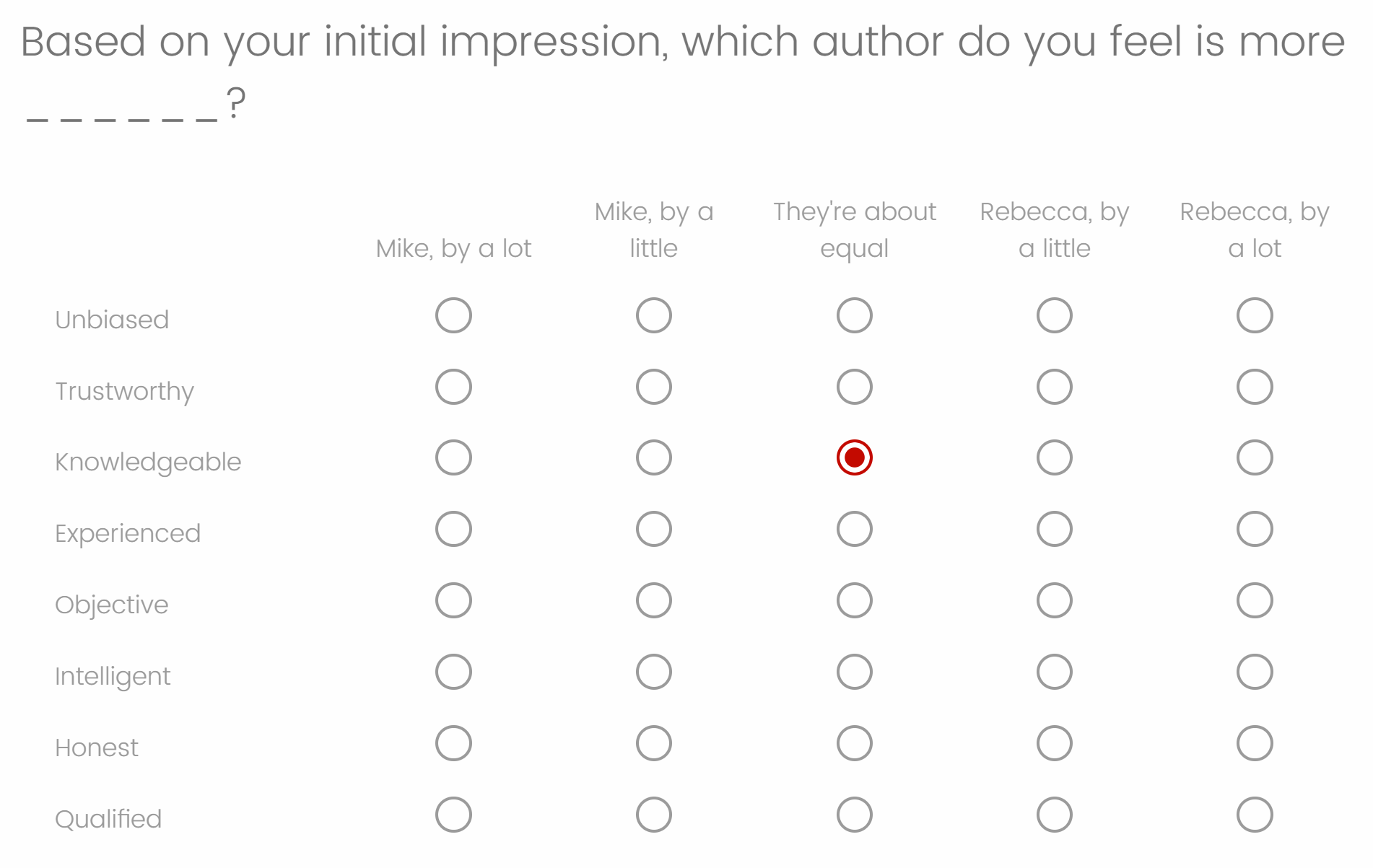
**

**
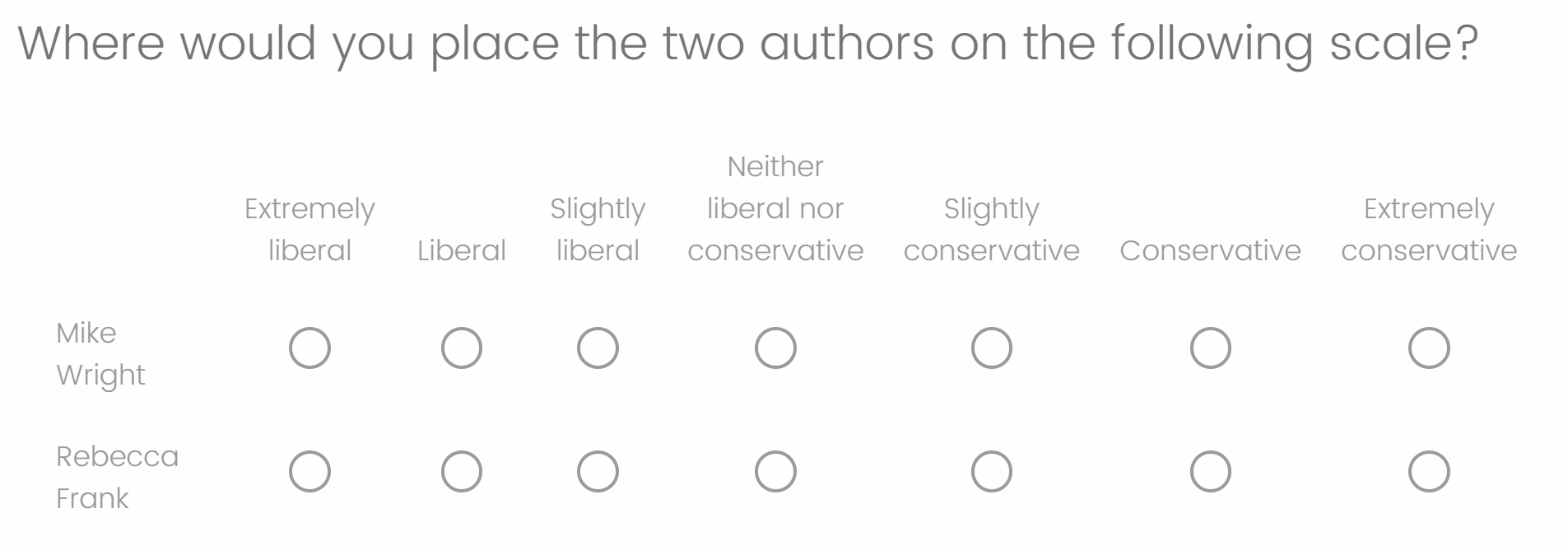
**

**
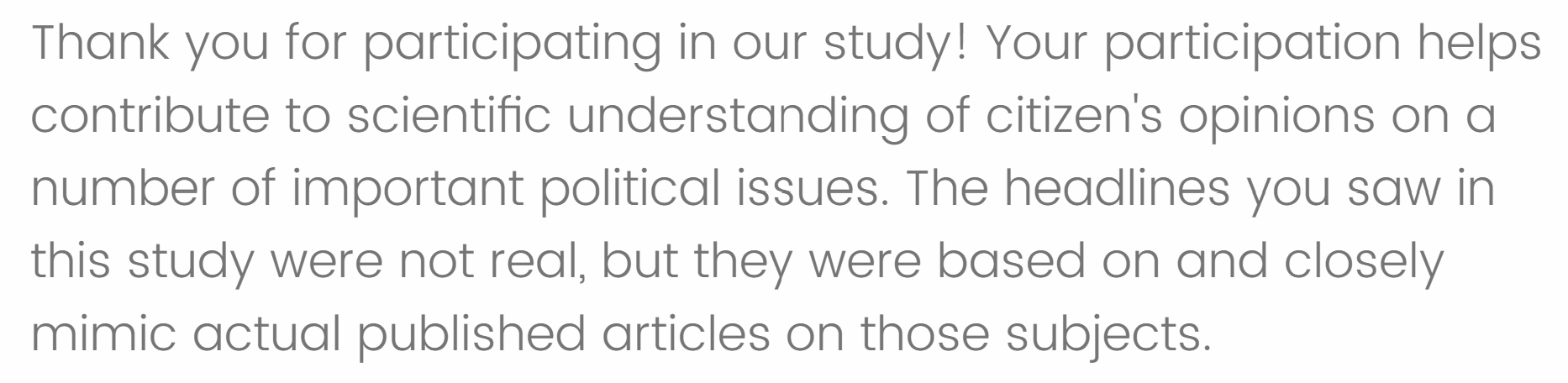
**
